# Elevating theranostics: The emergence and promise of radiopharmaceutical cell-targeting heterodimers in human cancers

**DOI:** 10.1002/ird3.62

**Published:** 2024-04-06

**Authors:** Claudia Chambers, Broc Chitwood, Charles J. Smith, Yubin Miao

**Affiliations:** 1 Molecular Imaging and Theranostics Center, Columbia, Missouri, USA; 2 Research Division, Harry S. Truman Memorial Veterans’ Hospital, Columbia, Missouri, USA; 3 Department of Chemistry, University of Missouri, Columbia, Missouri, USA; 4 Department of Radiology, University of Missouri School of Medicine, Columbia, Missouri, USA; 5 University of Missouri Research Reactor Center, University of Missouri, Columbia, Missouri, USA; 6 Department of Radiology, University of Colorado Denver, Aurora, Colorado, USA

**Keywords:** heterodimer, heterobivalent, hybrid peptide, PSMA, GRPR, α_v_β_3_, SST, MSH

## Abstract

Optimal therapeutic and diagnostic efficacy is essential for healthcare’s global mission of advancing oncologic drug development. Accurate diagnosis and detection are crucial prerequisites for effective risk stratification and personalized patient care in clinical oncology. A paradigm shift is emerging with the promise of multi-receptor-targeting compounds. While existing detection and staging methods have demonstrated some success, the traditional approach of monotherapy is being reevaluated to enhance therapeutic effectiveness. Heterodimeric site-specific agents are a versatile solution by targeting two distinct biomarkers with a single theranostic agent. This review describes the innovation of dual-targeting compounds, examining their design strategies, therapeutic implications, and the promising path they present for addressing complex diseases.

## 1 | INTRODUCTION

**FIGURE 1:**
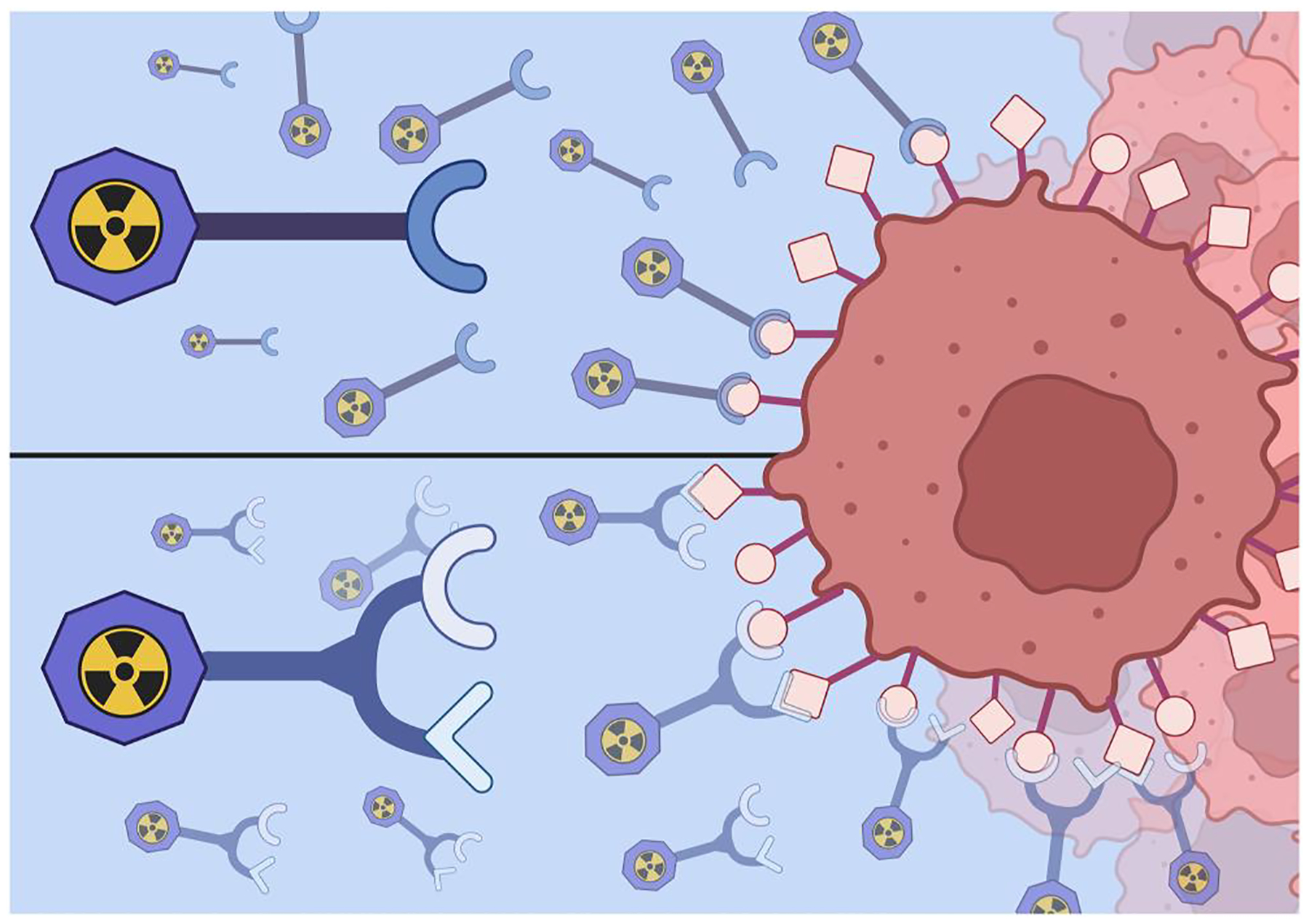
Basic design comparison of a monovalent radiopharmaceutical (top) compared to a bivalent radiopharmaceutical (bottom) targeting receptors on a cancer cell. Created with BioRender.com.

A groundbreaking approach is emerging in personalized medicine, which employs multimeric peptide-based radiopharmaceuticals for diagnosis and possible therapy of human disease, including cancer. Traditionally, researchers have designed site-directed radiotracers based on a monovalent approach that targets only one biomarker [1, 2]. These radiopharmaceuticals are composed of a solitary targeting vector (such as bioactive peptides, small molecules, nanoparticles, antibodies, or antibody fragments), a pharmacokinetic modifier, a bifunctional chelating agent (BFCA), and a radiometal appropriate for theranostic use (Figure 1). While these radiotracers have exhibited significant success, redirecting to a heterobivalent approach is thought to improve pharmacological properties of the radiotracer and to increase the potential theranostic payload [3–11].

Peptide-based cell targeting radiopharmaceuticals are designed to minimize harm to healthy tissues, distinguishing them from conventional chemotherapy methods for treatment of disease [12]. This synthetic peptide approach is a straightforward process allowing for customization to specifically target biomarkers primarily found on tumor cells in certain human malignancies while sparing normal tissue cells. Additionally, peptide-based radiotracers possess an improved ability to penetrate tumors and their vasculature [13]. Through structural modifications, they can be rapidly eliminated from the bloodstream and non-target tissues to improve pharmacological efficacy. Therefore, ensuring the molecular peptide arrangement is properly configured to have ideal metabolic and biological half-life is essential [14, 15]. To improve the effectiveness of peptide-based radioligands for molecular imaging or targeted radiotherapy (TRT), appropriate radiometals should be chosen. Nuclear characteristics such as half-life, decay mode, and emission profile (Table 1) are important when choosing a radiometal for diagnostic or therapeutic use. For diagnostic imaging, it is important to select either a positron-emitting radioisotope for positron emission tomography (PET) or a gamma-emitting radionuclide for single-photon emission tomography (SPECT). When choosing a radionuclide for TRT, beta or alpha-emitting isotopes are necessary. In addition, the BFCA must have suitable coordination chemistry for the radionuclide of choice to ensure stability of the metal for preclinical and clinical investigations.

A limitation of monovalent cell-targeting agents is their ability to only target a single biomarker or receptor. While radiotracers targeting singular cell surface receptors have demonstrated significant success, these agents lack the versatility to accommodate the possible complexities of malignancies [16]. The inability of these tracers to adapt for diverse cancer stages and varying target expression could limit personalized patient approaches.

There are many supporting factors in using a bivalent multi-targetable approach. For example, utilizing a single agent capable of targeting multiple biomarkers has the potential to enhance sensitivity in detection through improved binding affinity [7–10]. Tumor microenvironment complexity shows diverse levels and varieties of receptor site expression, presenting a challenge for effective targeting. Biomarkers within these malignancies are dynamic, variably expressed throughout the many stages of tumor development, and even differ between individual patients. By directing a single agent toward multiple known biomarkers, research suggests that a wider range of patients can be encompassed, accounting for expression profiles at all stages [17]. This approach also offers enhanced targeting specificity and theranostic utility enabling precise staging, tailored treatment design, and potential improvements in patient outcomes and quality of life.

This article navigates the complexities of designing and harnessing the power of novel radiotracers that harmonize the dual receptor-targeting actions of these emerging theranostic radiopharmaceuticals for potential usage in clinical applications. This involves understanding the evolution of these synthetic peptide analogs that are optimized for in vivo stability, affinity, specificity, and pharmacokinetic profile. Additionally, advancements in radiochemistry and chelation chemistry have enabled the fine-tuning of these peptides for radiolabeling with various radionuclides, catering to diverse medical applications within nuclear oncology.

## 2 | SINGLE RECEPTOR CELL-TARGETING AGENTS (MONOVALENT RADIOTRACERS)

### 2.1 | Somatostatin receptor-targeting radiotracers

Somatostatin (SST) cell-targeting radiotracers have captivated researchers for over 3 decades. Somatostatin is a neuropeptide hormone that is expressed largely in the central and peripheral nervous system. Somatostatin has two native cyclic forms, SST-14, and SST-28. This hormone is produced on delta cell membranes in high quantities of human tissues and malignancies, the highest of which being in the gastrointestinal tract, pancreas, hypothalamus, and central nervous system. Somatostatin also serves as a neuromodulator and neurotransmitter in the central nervous system, affecting neuroendocrine (NE), motor, and cognitive functions, as well as influencing the synthesis of growth factors. Somatostatin plays a crucial role in inhibiting both endocrine and hormone secretion via the five known *G* protein coupled receptors SSTR1–5.

**FIGURE 2:**
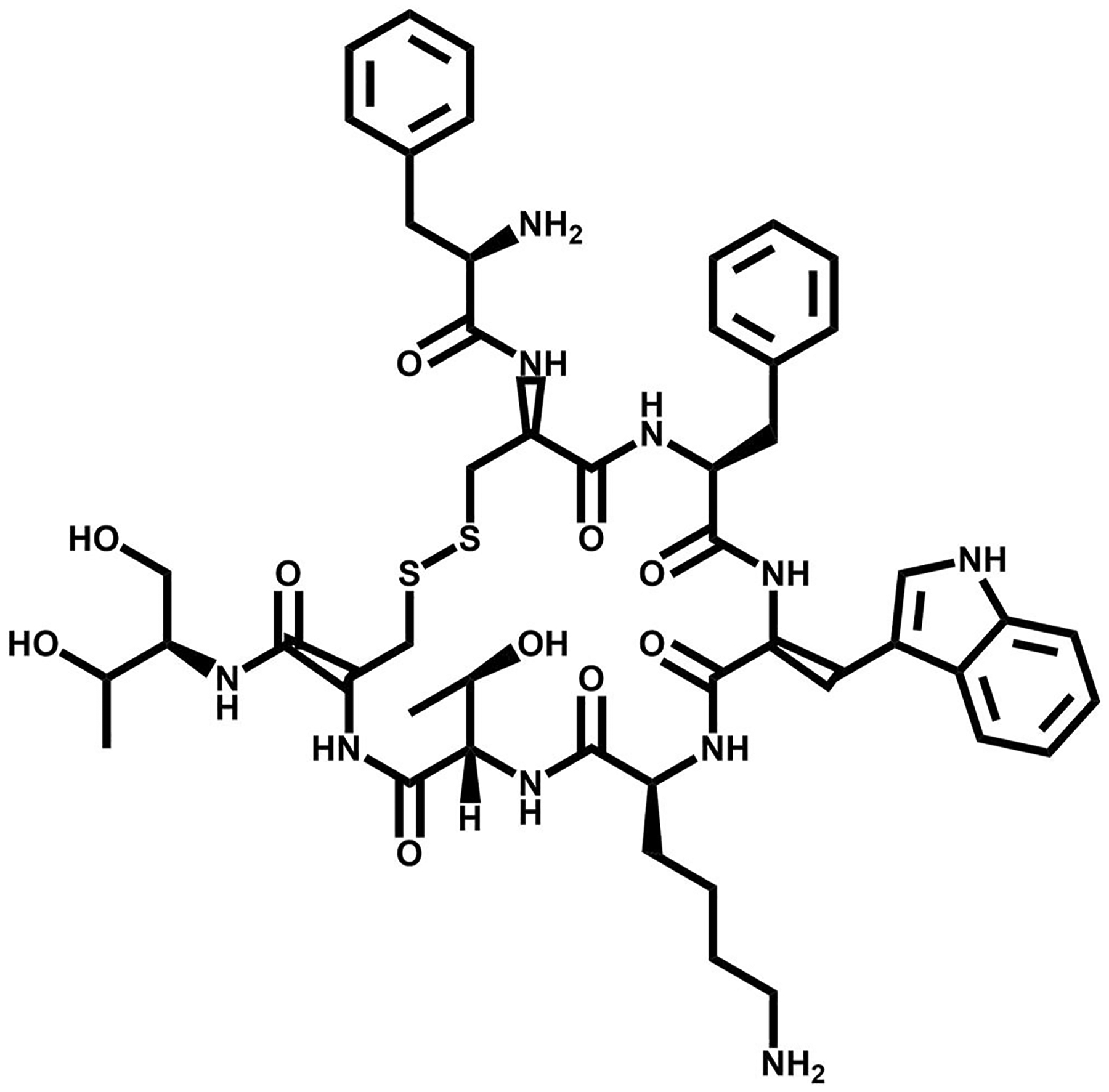
The chemical structure of octreotide, a somatostatin (SST) targeting vector.

The most clinically useful subtype, SSTR2, is expressed in elevated levels on human neuroendocrine tumors (NET) and neuroendocrine carcinomas [18–20]. The elevated levels of SST receptors on NE neoplasms, as well as SST’s antiangiogenic effects has led to the development of radiotheranostic analogs of SST. Peptide receptor radionuclide therapy derivatives have been targeting SST based on the biologically-active tetrapeptide Phe-Trp-Lys-Thr for receptor recognition [21, 22]. While the native hormones SST-14 and SST-28 exhibit significant affinity for all SST receptor subtypes, shorter analogs exclusively engage with the initial subgroup of receptor subtypes SST2, SST3, and SST5. Optimizing analogs of SST has been critical to improve stability, receptor recognition, and biological activity to the different receptor subtypes. Integrating D-amino acids for increased stability onto SST’s minimal amino acid sequence has resulted in an octapeptide. This octapeptide is characterized by the active core sequence Phe-D-Trp-Lys-Thr, which forms a six-membered ring via a single disulfide bridge (Figure 2). This peptide is recognized as Octreotide^®^ [23].

In the realm of PET and SPECT molecular imaging, efforts have been made to develop radiotracers that particularly target SST2 positive NET. These radiotracers utilize different radiolabeling strategies and linkers, often based on the octreotate/octreotide peptide structure. The evolution of SST monovalent radiotracers is detailed, beginning with Lamberts et al.’s 1989 study using ^123^I-labeled Try^3^-octreotide [D-Phe-Cys-Phe-D-Trp-Lys-Thr-Cys-Thr(ol)], also known as TOC [24], to visualize endocrine-related tumors [25]. Notable advancements include employing chelators such as diethylenetriaminepentaacetic acid (DTPA) and DOTA (1,4,7,10-tetraazacyclododecane-1,4,7,10-tetraacetic acid) to label radiometals for therapeutic use like ^90^Y and ^177^Lu. This paved the way for Peptide receptor radionuclide therapy utilizing agonists such as DOTA-TOC and DOTA-TATE [26, 27]. United States Food and Drug Administration (FDA) approval for the diagnosis and treatment of NETs of three radiotracers, 177Lu-DOTATATE (LUTATHERA^®^), ^68^Ga-DOTATATE (NETSPOT^®^), and 111In-octreotide (SANDOSTATIN^®^), have marked a significant milestone in the field of theranostics [28]. As a result, a successful theranostic approach targeting these receptors has emerged and has shown positive results in reducing tumor growth and improving patient survival. The success of SST-targeted peptide-based agents has paved the way for exploring similar approaches for other receptor systems, such as Prostate Specific Membrane Antigen (PSMA), melanocortin-1 receptor, bombesin, and integrin complex α_v_β_3_.

Researchers have successfully modified the octreotide structure to convert it from an agonist to an antagonist via structural inversion of chirality. The conversion was explored based upon the notion that antagonists bind to a higher number of receptors than agonists in preclinical evaluations, making them a potentially better candidate for tumor targeting. From the very first preclinical evaluation conducted by Ginj et al. [29] the superiority of radiolabeled SST antagonists over agonists was established.

### 2.2 | Prostate specific membrane antigen targeting radiotracers

Several targeted radiolabeled peptides and small molecules have been innovated to cater to both imaging and therapy requirements for prostate cancer (PCa). One effective approach is targeting PSMA, also known as folate hydrolase I or glutamate carboxypeptidase II. PSMA is a 750-amino acid integral membrane protein predominantly found in the neovasculature of most solid tumors including renal, gastric, pancreatic, breast, brain, and colorectal carcinomas. However, it remains negligible or absent in the normal vascular endothelium of collateral tissues [4, 30]. The ability of PSMA to be rapidly internalized via clathrin-coated pits, coupled with a high incidence of expression on various neoplasias makes this a favorable target in oncologic drug research. PSMA targeting motifs have played a crucial role as both a diagnostic and therapeutic target for PCa research and more recently clinical applications.

PSMA’s primary expression in PCa research is observed in the epithelium of prostate tumor cells and, to a lesser extent, in the vasculature of PCa itself. Remarkably, the density per cancer cell reaches approximately 10^6^, a figure approximately 1000-fold greater than normal PSMA-expressing tissues located in the kidney, brain, and small intestine [31]. While primarily confined to PCa cell epithelium, the behavior of PSMA changes as the disease progresses to androgen-independent, metastatic stages [32]. This alteration includes upregulation and translocation of PSMA from internal organelles to the cell surface. Notably, upregulated receptor expression has been observed in metastatic lesions located in lymphatic tissue, bones, and lungs [33]. PSMA’s high overexpression on PCa cells, especially advanced-stage carcinomas, as well as swift internalization, make it a suitable target for nuclear imaging and TRT.

**FIGURE 3:**
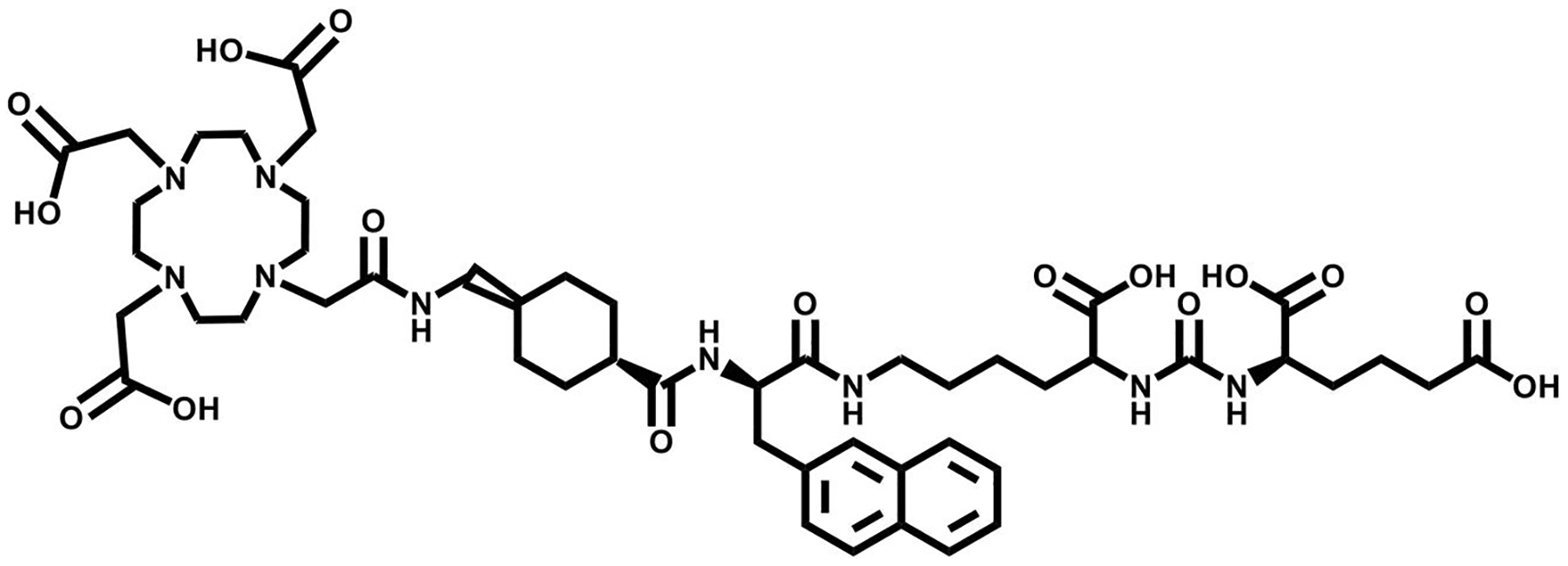
The chemical structure of PSMA-617, a prostate specific membrane antigen (PSMA) targeting vector.

Motifs target PSMA through a zinc-cofactor binding site via small molecule enzyme inhibitors. Among these are glutamate-conjugated ureas, thiols, and phosphorus compounds. One of the first FDA-approved imaging agents in 1996 is the Indium-radiolabeled antibody ^111^In-DTPA-7E11-C5.3, better known as ProstaScint^®^ [34]. While this radiotracer has been widely used for targeting PSMA, limitations of using an antibody-based agent have been challenging. These shortcomings, such as inadequate penetration of solid tumors and slow clearance rates from blood serum and collateral tissues, are being mitigated with the use of radiolabeled peptides and small molecules as alternatives [35]. Glu–urea–glu-based molecules have been designed as an efficient solution in detecting both primary and advanced PCa metastasis. Urea-based ligands showed the most promising results in preclinical trials, while phosphorous and thiol-based ligands showed limited opportunities for clinical use due to poor pharmacokinetic profiles. For example, DUPA (2-[3-(1,3-dicarboxypropyl)-ureido]pentanedioic acid) has been previously labeled with ^18^F, ^64^Cu, ^68^Ga, and ^86^Y for PET molecular imaging [36]. Of these derivatives, ^68^Ga-PSMA-11 (Locametz^®^), was pioneered by Afshar-Oromieh and colleagues in 2012. After Locametz’s FDA approval in December of 2020 as well as its endorsement of clinical use by both University of California Los Angeles and University of California San Francisco, interest to clinically use the DUPA derivative for TRT peaked [37, 38]. In 2022, ^177^Lu-PSMA-617 (Pluvicto^®^) (Figure 3) was FDA approved for treatment of late stage PCa, showing a reduction ≥50% of PSA levels in PCa patients after their first treatment [39, 40]. Other PSMA targeting small molecules that have demonstrated success in staging PCa in patients have been extensively reported [41–43].

### 2.3 | Gastrin releasing peptide receptor targeting radiotracers

The neuropeptide bombesin peptide (BBN) is a 14-amino acid peptide with a C-terminal carboxyamide. The Gastrin Releasing Peptide Receptor (GRPR) is a *G* protein-coupled receptor that is well known in the BBN family [44]. This 27-amino acid peptide can be found naturally in the nervous system and the gastrointestinal tract stimulating hormone release and is also overexpressed in various neoplasias including prostate, breast, pancreas, and lung, making it an attractive theranostic target for oncology [45]. Currently, many GRPR radioligands are in preclinical and clinical investigations for the diagnosis and therapy of PCa. The expression of GRPR correlates with tumor grade stage, and other factors [46]. Prostatic tumor cells exhibit elevated levels of GRPR expression, whereas normal prostate tissue demonstrates minimal presence of this receptor [46–50]. GRPR monovalent targeting ligands gained prominence in earlier studies due to their swift internalization within GRP-positive malignancies and their potential for clinical applications. Both BBN antagonist and agonist analogs labeled with various radionuclides have been developed for the purpose of imaging tumors that express GRPR and for TRT [51, 52].

First-generation bombesin analogs have demonstrated prolonged retention within targeted cells due to their internalization properties, suggesting potential enhanced in vivo uptake. Various radiotracers have been developed and evaluated including [^68^Ga/^177^Lu/^111^In]-AMBA [53–55], ^18^F-labeled variations including NOTA-8-Aoc-BBN(7–14)NH_2_ [56] and Carlucci’s [^18^F]FAl-labeled lanthionine-stabilized BBN analogs [57]. Lane et al. synthesized a series of [^64^Cu]NO2A-(X)-BBN(7–14)NH_2_ agonists where X = AMBA was reported to have superior accumulation, retention, and clearance results in vivo in tumor bearing mice [58]. While the agonists overall demonstrated satisfactory performance, their adverse effects on the gastrointestinal system limited further development.

**FIGURE 4:**
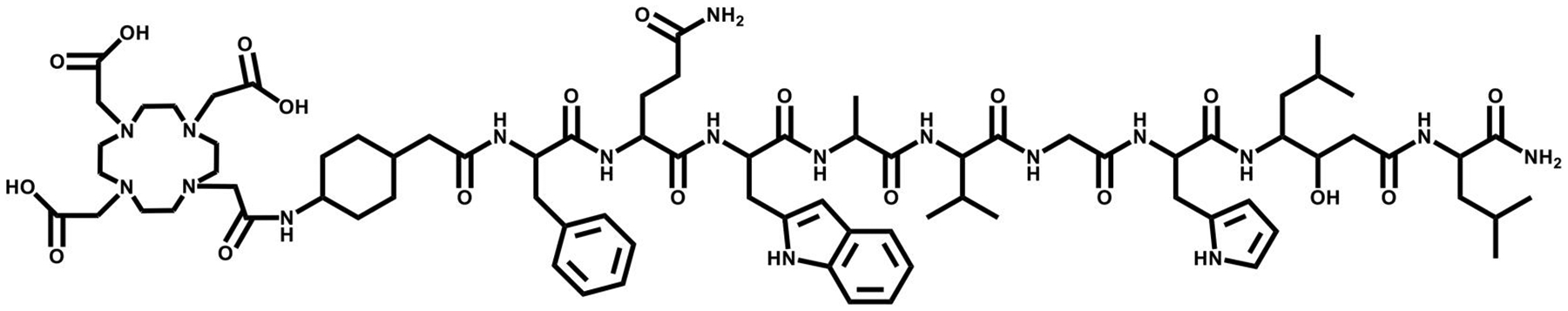
The chemical structure of DOTA-RM2, a gastrin releasing peptide receptor (GRPR) targeting vector.

A shift of strategy began for BBN analogs when a GRP non-internalizing antagonist showed favorable drug disposition characteristics [59]. The second generation development of GRPR radiotracers began when SST2 antagonists demonstrated an increased cell targeting and retention compared to SST2 agonists. Mansi and colleagues synthesized an RM1 (DOTA-Gly-aminobenzoic acid-D-Phe-Gln-Trp-Ala-Val-Gly-His-Sta-Leu-NH_2_) antagonist and the In-111 labeled radiotracer to the agonist ^111^In-AMBA [54]. ^111^In-RM1 demonstrated superior results propelling a successful study with ^68^Ga-RM1. Currently, a radiopharmaceutical for RM2 (DOTA-4-amino-1-carboxymethyl-piperidine-D-Phe-Gln-Trp-Ala-Val-Gly-His-Sta-Leu-NH_2_) (Figure 4) derivative is undergoing evaluation for approval by the FDA for the treatment of patients with metastatic, castration-resistant, PCa [60, 61]. Studies in four patients who have received ^68^Ga-RM2 for PET/CT and ^177^Lu-RM2 for therapy demonstrate high tumor uptake, optimal metabolic clearance/uptake in benign tissues, and no observable side effects. Gastrin Releasing Peptide Receptor + cells also did not show changes in relative expression after being exposed to up to 10 Gy of external beam radiation [62].

Gastrin Releasing Peptide Receptor antagonists offer improved pharmacokinetics compared to agonist radioligands. Although they do not trigger receptor activation upon binding, these antagonists effectively target and remain in GRPR-expressing cancer lesions while quickly clearing from normal organs in both animals and humans [63]. Numerous GRPR ligands have demonstrated successful results in targeting PCa.

### 2.4 | α_v_β_3_ targeting radiotracers

Integrins are glycoproteins consisting of α and β subunits maintaining critical roles in various cellular processes including angiogenesis, cell attachment, and interactions with the extracellular matrix. Angiogenesis allows new blood vessels to form from existing ones acting as a gateway for the tumor cells to transition from dormant to malignant and metastatic. Additionally, it aids in the breakdown of extracellular and interstitial matrices by activation of the matrix metalloproteinase and plasmin. A well-established integrin, α_v_β_3_, plays a suspected significant role in the development of multiple cancer metastasis [64]. It is displayed in multiple tumor environments such as malignant melanoma, osteosarcoma, glioblastoma, breast cancer, and PCa. Researchers have coined the minimal amino acid sequence Arg-Gly-Asp (RGD) as the targeting peptide motif for α_v_β_3_ receptors overexpressed on tumor and neovasculature [65, 66]. α_v_β_3_ is comparatively absent in normal tissues despite mild expression on active endothelial cells and newly formed vessels. α_v_β_3_’s comparatively high expression in tumors allows for RGD analogs to target only tissues of interest [67, 68].

The RGD triad has been extensively researched for numerous peptides binding to α_v_β_3_ receptors. RGD provides a more than adequate site for conjugation with multiple radionuclides including that is, ^99m^Tc, ^111^In, ^68^Ga, ^18^F, and ^64^Cu [69]. Monomeric radiotracers have demonstrated the ability to specifically target α_v_β_3_ integrin efficiently, allowing for the potential of diagnostic and therapeutic approaches for cancer [70]. Both linear and cyclic varieties have been researched in attempts to improve binding affinity and pharmacokinetic properties [71, 72].

**FIGURE 5:**
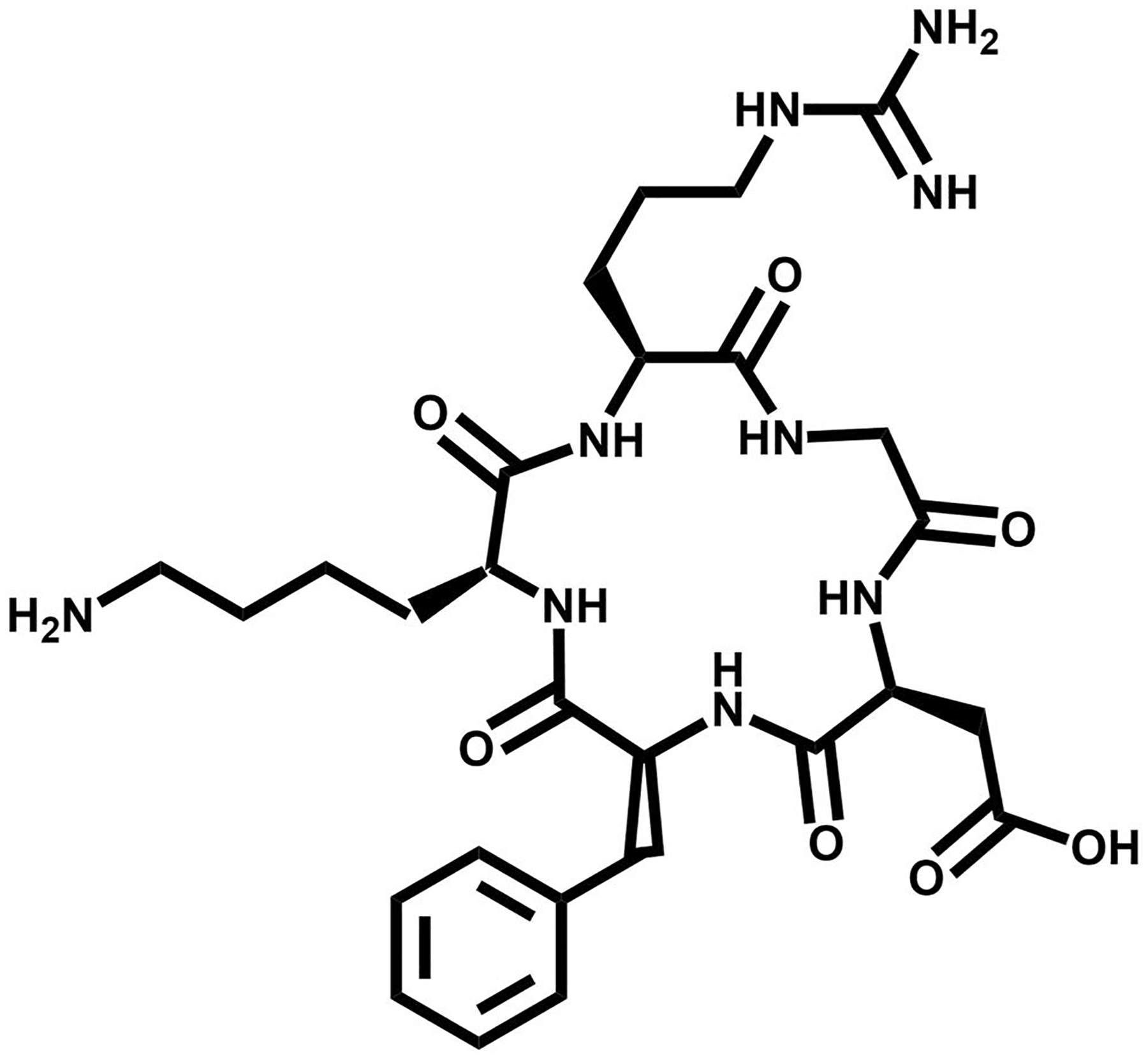
The chemical structure of c[RGDfK], an α_v_β_3_ targeting vector.

Several RGD compounds have demonstrated potential for theranostic applications including the cyclodecapeptide scaffold regioselectivity addressable functionalized template (RAFT) [73, 74]. This scaffold displays four c[RGDfK] ligands with a DOTA complex to facilitate radiolabeling with various radionuclides. Among these radionuclides, ^90^Y and ^177^Lu for TRT of α_V_β_3_-positive tumors were utilized with DOTA, demonstrating high specific tumor accumulation with 10:1 tumor/muscle ratio at 1 h post injection (p.i.) [75]. A cyclam chelator was then replaced on the RAFT scaffold to enable ^64^Cu PET imaging of α_V_β_3_ malignancies [76]. Theranostic RAFT(c[-RGDfK-] 4)–radionuclide conjugates (Figure 5) proved efficient to combine both in vivo cancer diagnostic and therapy in mice in several types of cancers with high tumor penetration and no toxicity [77]. To date, RGD peptides combined with radionuclides have been intensively studied as radiotracers for tumor imaging, with ongoing clinical trials for the PET/CT imaging in various cancers.

### 2.5 | Melanocortin-1 receptor targeting radiotracers

α-Melanocyte-Stimulating Hormone (α-MSH) is a peptidic pituitary hormone in the family of melanocortin. The 13 amino acid neuropeptide is secreted by melanocytes in response to ultraviolet light and has been extensively studied regarding its interaction with G-protein coupled receptors as well as inducing melanin production. While the primary role of α-MSH is melanin synthesis post-ultraviolet light exposure, it also plays a key role in processes such as repairing DNA damage, production of free radicals, and promoting cell proliferation. Melanocortin-1 receptor (MC1R) is the primary target for α-MSH binding and is found to be significantly overexpressed on primary and metastatic melanoma in comparison to the levels observed in normal cells [78–83]. Thus, α-MSH and its analogs are suitable for MC1R-targeted radiopharmaceutical design and development [84–90].

**FIGURE 6:**
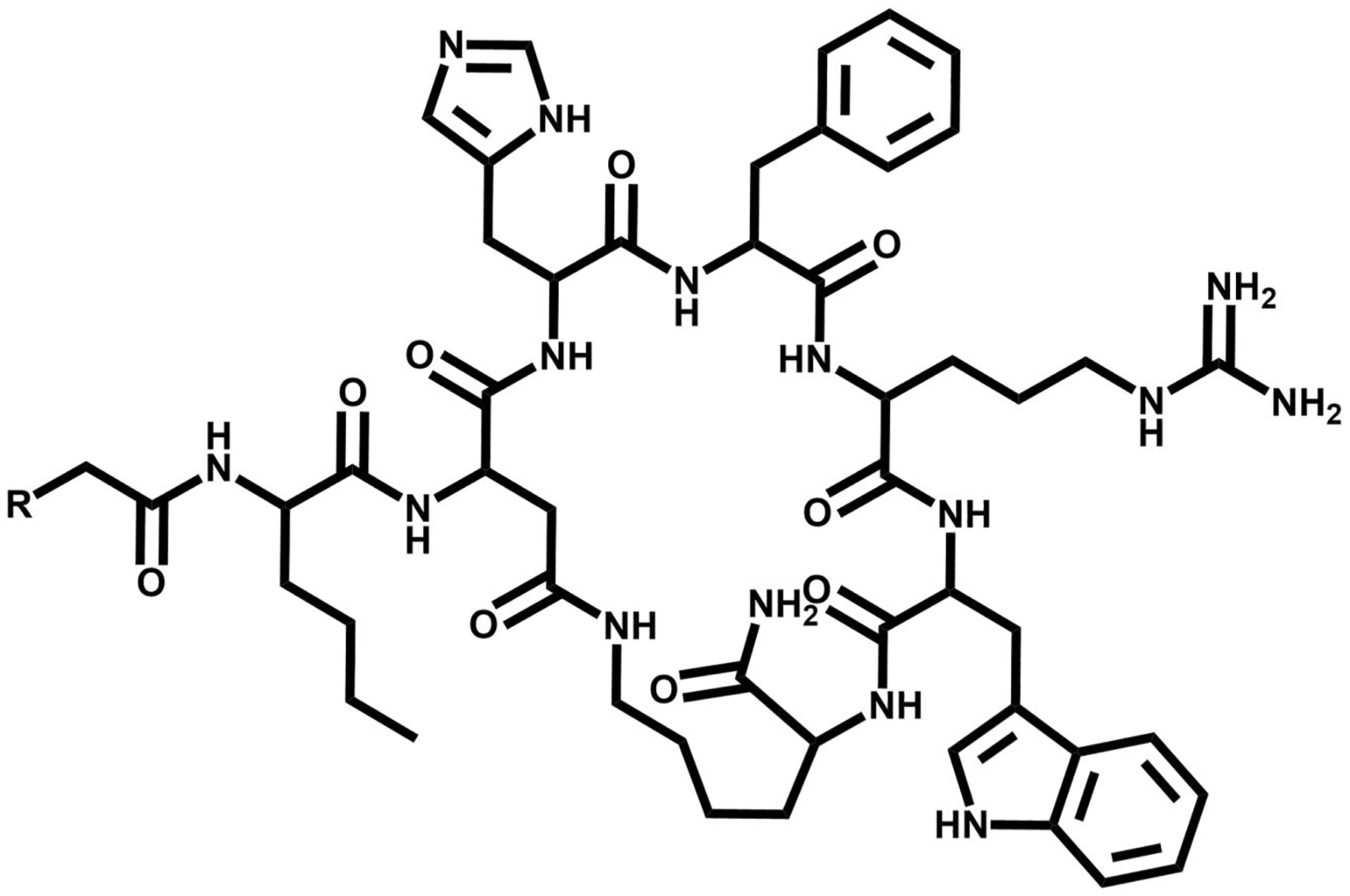
The chemical structure of Nle-CycMSH_hex_, an MC1R targeting vector where *R* denotes complexing agent.

Naturally occurring α-MSH peptide has a short biological half-life in vivo. Synthetic analogs of α-MSH have been created to overcome this limitation as well as to enhance targeting capabilities. Various modified forms of α-MSH have been developed to enhance biological stability and targeting, including DOTA-[Nle^4^, D-Phe^7^]α-MSH (DOTA-NDP-MSH), DOTA-NAPamide (Ac-Nle-Asp-His-D-Phe-Arg-Trp-Gly-Lys(DOTA)-NH_2_), DOTA-ReCCMSH, DOTA-GlyGlu-CycMSH, and DOTA-Nle-CycMSH_hex_ (Figure 6). Many of these peptides have been successfully labeled with various radiometals such as^64^Cu, ^68^Ga, ^18^F, ^111^In, ^99m^Tc, etc [91]. Miao and colleagues developed another class of MC1R-targeted peptide radiopharmaceutical, GGNle-CycMSH_hex_, for melanoma imaging and therapy. Yang et al. conducted initial tests with ^68^Ga-DOTA-GGNle-CycMSH_hex_ which demonstrated successful detection of human metastatic melanoma lesions, indicating the clinical relevance of MC1R for imaging and potential therapy [80]. Looking into MC1R TRT peptides, Qiao et al. explored the potential of ^67^Cu-NOTA-PEG_2_Nle-CycMSH_hex_ in B16/F10 melanoma bearing C57 mice which displayed favorable biodistribution properties including high tumor uptake (24.10±1.83%ID/g) at 2 h p.i[92]. These ligands, among others, hold promise as platforms for molecular imaging of melanoma, offering enhanced targeting capabilities and potential applications in melanoma-related research and clinical practice. Overall, the efforts to modify and optimize α-MSH and its analogs have significantly expanded their utility in melanoma-targeted imaging and therapeutic strategies.

## 3 | DUAL RECEPTOR CELL-TARGETING AGENTS (BIVALENT RADIOTRACERS)

### 3.1 | α_v_β_3_-gastrin-releasing peptide receptors cell-targeting radiotracers

Today, research groups have begun to pivot away from the usage of monovalent radioligands, as emerging research into simultaneous, multi-receptor, cell-targeting using heterodimeric conjugates shows potential in enhancing diagnostic capabilities and therapeutic efficacy. Two biomarkers of interest for heterodimeric use are both expressed in very high numbers on the surfaces of PCa cells, α_v_β_3_ and GRPR. Targeting both biomarkers with a single radiotracer has been shown to improve retention and accumulation in tumors for TRT and molecular imaging. Along with enhanced pharmacological properties, a multivalent peptide for PCa may also be able to reach a larger cohort of patients [3–6, 11]. This is potentially due to receptor density shift ranging in an order of magnitude when compared to either the GRPR or α_v_β_3_ monovalent species alone. Heterodimers that target both α_v_β_3_ and GRPR have been shown to be superior to monovalent GRPR or α_v_β_3_ [93–97]. For example, using a peptide that allows binding of multiple receptors has shown increased ability to be imaged in cases where one of the receptors is limited by low expression of the targeted biomarker (Table 2).

**FIGURE 7:**
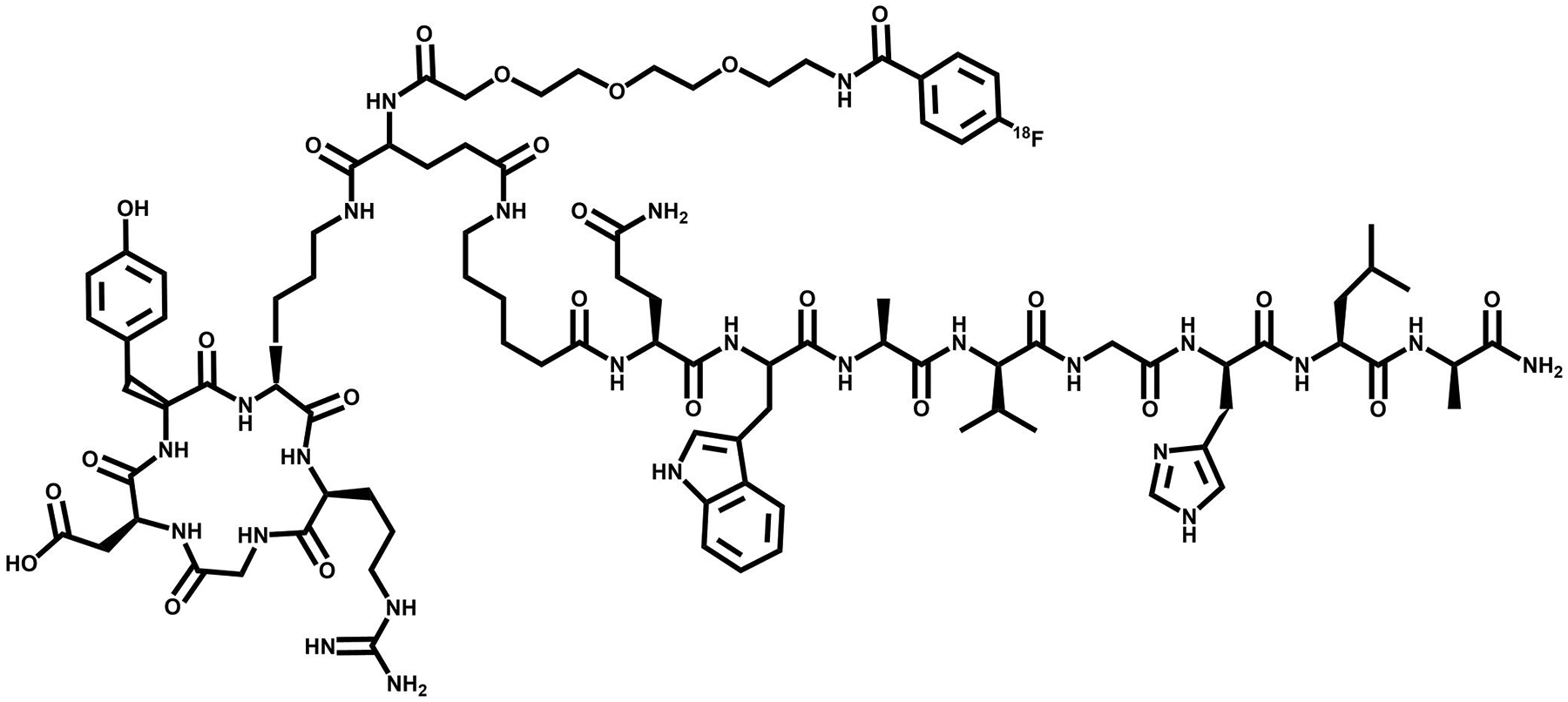
Structure of 18F labeled FB-PEG2-Glu-RGD-BBN, a heterobivalent α_v_β_3_-GRPR targeting peptide

**FIGURE 8:**
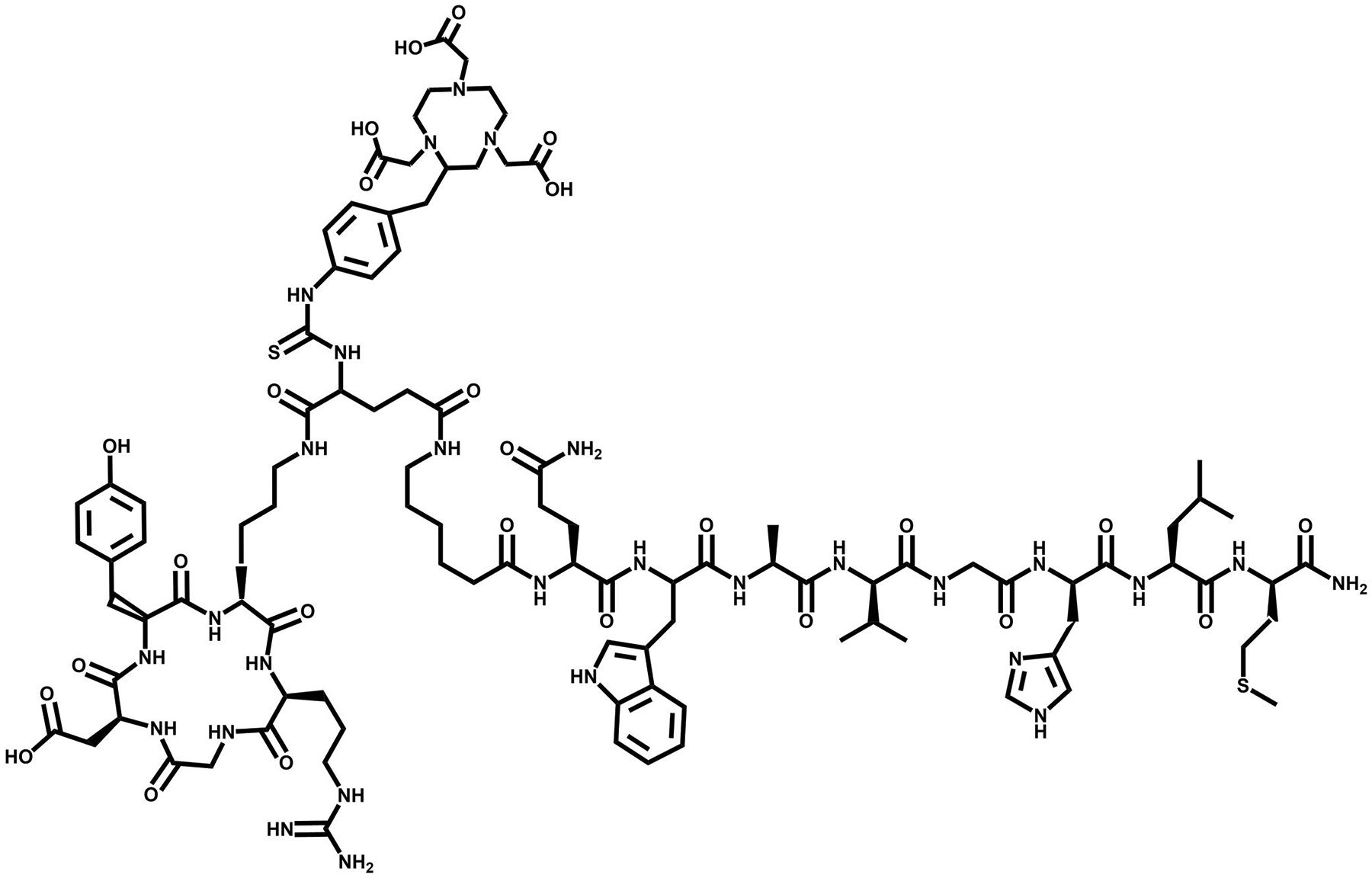
Structure of NOTA-RGD-BBN, a heterobivalent α_v_β_3_-GRPR targeting peptide.

One of the first research groups to target both GRPR and α_v_β_3_ receptors simultaneously was the Chen lab [99]. BBN and RGD were linked with a glutamate amino acid residue to formulate RGD-BBN (Figure 7), which was successfully labeled with ^18^F and showed promising results in vitro and in vivo [93]. Subsequent to these promising results, NOTA conjugation of the bivalent molecule was evaluated. Production of the ^68^Ga radiolabeled NOTA-RGD-BBN was substantially more streamlined in comparison to the 18F first generation bivalent molecule and demonstrated more favorable in vivo results [93]. (Figure 8) Biodistribution results of ^68^Ga-NOTA-RGD-BBN in PC3 xenografted tumor-bearing mice were higher (5.26 ± 0.32%ID/g) at 1 h in comparison to the 18F-RGD-BBN uptake (4.41%ID/g) [98]. To elaborate upon the dual GRPR-α_v_β_3_ targeting structure, PEG_3_-Glu-RGD-BBN was created by attaching a polyethylene glycol (PEG) spacer and 18F-SFB as a synthon to improve the pharmacokinetic properties and enhance ^18^F labeling yields [100, 101]. Upon successful radiolabeling with a radiochemical purity of ≥99%, the receptor cell binding affinities of ^18^F-FB-PEG_3_-Glu-RGD-BBN were observed to align with their respective monomers, Aca-BBN(7–14) and c(RGDyK) [98]. Administration of a blocking agent resulted in complete block of both the integrin and GRPR, confirming the specificity and selectivity of this compound toward both biomarkers of interest. Positron emission tomography images of mice bearing PC3 tumors displayed significant tumor contrast for up to 120 min p.i., accompanied by a decrease in kidney uptake during the earlier time points. This trend suggests that the compound is primarily cleared through the renal-urinary excretion pathway. Further investigations utilizing positron-emitting agents involved the use of various cell lines. In vivo studies were conducted using breast cancer cell models, namely T47D (GRPR+/low α_v_β_3_) and MDA-MB-435 (GRPR-/α_v_β_3_+), with ^18^-F-FB-PEG_3_-RGD-BBN, ^68^Ga-NOTA-RGD-BBN, and ^64^Cu-NOTA-RGD-BBN [97]. PET imaging of each compound was evaluated and quantified to assess pharmacokinetic properties in the blood, liver, kidney, muscle, and tumors of tumor-bearing nude mice. While ^18^F-FB-PEG3-RGD-BBN exhibited lower tumor uptake at 1 h p.i. (T47D: 1.81 ± 0.34%ID/g; MDA-MB-435: 1.59 ± 0.65%ID/g) compared to the radiometalated conjugates, this conjugate demonstrated significantly lower background uptake in collateral organs [97]. In the T47D (GRPR+) cell line, both ^64^Cu-NOTA-RGD-BBN and ^68^Ga-NOTA-RGD-BBN exhibited tumor uptake at 1 h of 2.33 ± 0.59%ID/g and 2.78 ± 0.87%ID/g, respectively. Conversely, in the MDA-MB-435 (GRPR-) cell line, the tumor uptake at 1 h for ^64^Cu-NOTA-RGD-BBN and 68Ga-NOTA-RGD-BBN was 1.84 ± 0.44%ID/g and 2.24 ± 0.73%ID/g, respectively. Although 64Cu-NOTA-RGD-BBN displayed prolonged liver uptake, it also exhibited the highest liver and kidney accumulation. All of the positron-emitting radiotracers discussed herein effectively targeted the tumors of interest for diagnostic imaging, thus showing some potential for therapeutic applications. Therapeutic efficacy was evaluated using ^177^Lu-radiolabeled DO3A-RGD-BBN through biodistribution investigations [94]. In mice bearing PC3 xenografted tumors, accumulation of ^177^Lu-DO3A-RGD-BBN was measured at 5.88 ± 1.12%, 2.77 ± 0.30%, 2.04 ± 0.19%, and 1.18 ± 0.19% ID/g at 0.5, 2, 24, and 48 h p.i., respectively. Notably, rapid clearance from normal tissues led to elevated tumor-to-blood and tumor-to-muscle ratios, highlighting the potential clinical utility of the RGD-BBN theranostic agent.

In 2012, the radiolabeled compound 64Cu-NO2A-RGD-Glu-6-Ahx-BBN(7–14)NH_2_ underwent to comprehensive evaluation to assess its affinity for α_v_β_3_ and GRPR dual targeting [95]. This study compared CB-TE2A, NO2A, NO3A, and sarcophagine ligands, and variance caused by the slight change in the targeting vector effects changes in the biodistribution. Optimizing the pharmacokinetic modifiers while minimizing the background to produce ideal images. The study of the utilization of NO2A as a BFCA was suggested to mitigate the previously observed elevated kidney uptake associated with RGD/BBN conjugates, as reported by Liu [102–104]. In vivo investigations substantiated this proposition, demonstrating reduced kidney uptake. The uptake at 1 h in the tumor-bearing displayed a value of 4.65 ± 0.78%ID/g representing a higher kidney uptake than Liu’s compound of 3.06 ± 0.25%ID/g. Although the compound had higher initial uptake at the 24 h mark, kidney retention was 1.20 ± 0.36%ID/g [95]. This is slightly lower than 1.87 ± 0.41%ID/g at 20 h time point of the anionic analog, showing efficient clearance from the renal system. In addition to lower renal uptake, the new radioligand maintained binding to both biomarkers and achieving heightened radioligand uptake in PC3 xenografts. The study exhibited higher initial tumor uptake at 1 h 3.95 ± 1.04%ID/g compared to Liu’s 2.78 ± 0.56%ID/g and both showed an excellent ability to retain activity within tumor sites for several hours post injection [95]. Liver uptake represented in the heterodimeric 64Cu-NO2A-RGD-Glu-6-Ahx-BBN(7–14)NH_2_ displayed lower liver retention in comparison to the monomeric ^64^Cu-NO2A-6-Ahx-BBN(7–14)NH_2_ Notably, the radiolabeled conjugate exhibited rapid excretion through the urinary system and presented remarkable tumor-to-background ratios, demonstrating potential for effective imaging and targeted therapeutic applications.

Until the development of the heterodimeric compound [RGD-Glu-[NO2A]-6-Ahx-RM2], previous dual targeting conjugates aimed at α_v_β_3_ and GRPR primarily utilized an agonist-based BBN motif. Antagonistic analogs tend to exhibit enhanced uptake and retention in tumor tissue compared to agonist-based ligands, a phenomenon confirmed by Durkin et al [5], through preclinical investigations involving [RGD-Glu-[^64^Cu-NO2A]-6-Ahx-RM2]. To assess the pharmacokinetic properties of the peptide conjugate, [RGD-Glu-[^64^Cu-NO2A]-6-Ahx-RM2] was administered to CF-1 normal mice and tumor-bearing mice. Biodistribution analysis in normal mice over a 1 h period revealed rapid renal clearance, primarily noticeable at the 1 h mark. Negligible uptake was observed in GRPR + pancreas, as expected, and there was no uptake in GRPR-tissues, apart from some accumulation in the liver. Pancreatic uptake was anticipated and observed at the 1 h time point (4.70 ± 1.04%ID/g), with a substantial reduction (0.71 ± 0.08%ID/g) at 4 h. In PC3 tumor-bearing SCID mice, pharmacokinetics and excretion properties mirrored those observed in the normal mouse model. Tumor uptake was measured at 6.37 ± 1.23%ID/g at 4 h post-injection, and this accumulation was retained in the tumor (4.26 ± 1.23%ID/g) up to the 24 h time point [5]. Small animal PET images of the positron-emitting radiotracer in PC3 xenografted mice demonstrated high selectivity and sustained retention in the tumor, yielding high contrast diagnostic images consistent with biodistribution results. Minimal tracer accumulation was noted in collateral tissues.

**FIGURE 9:**
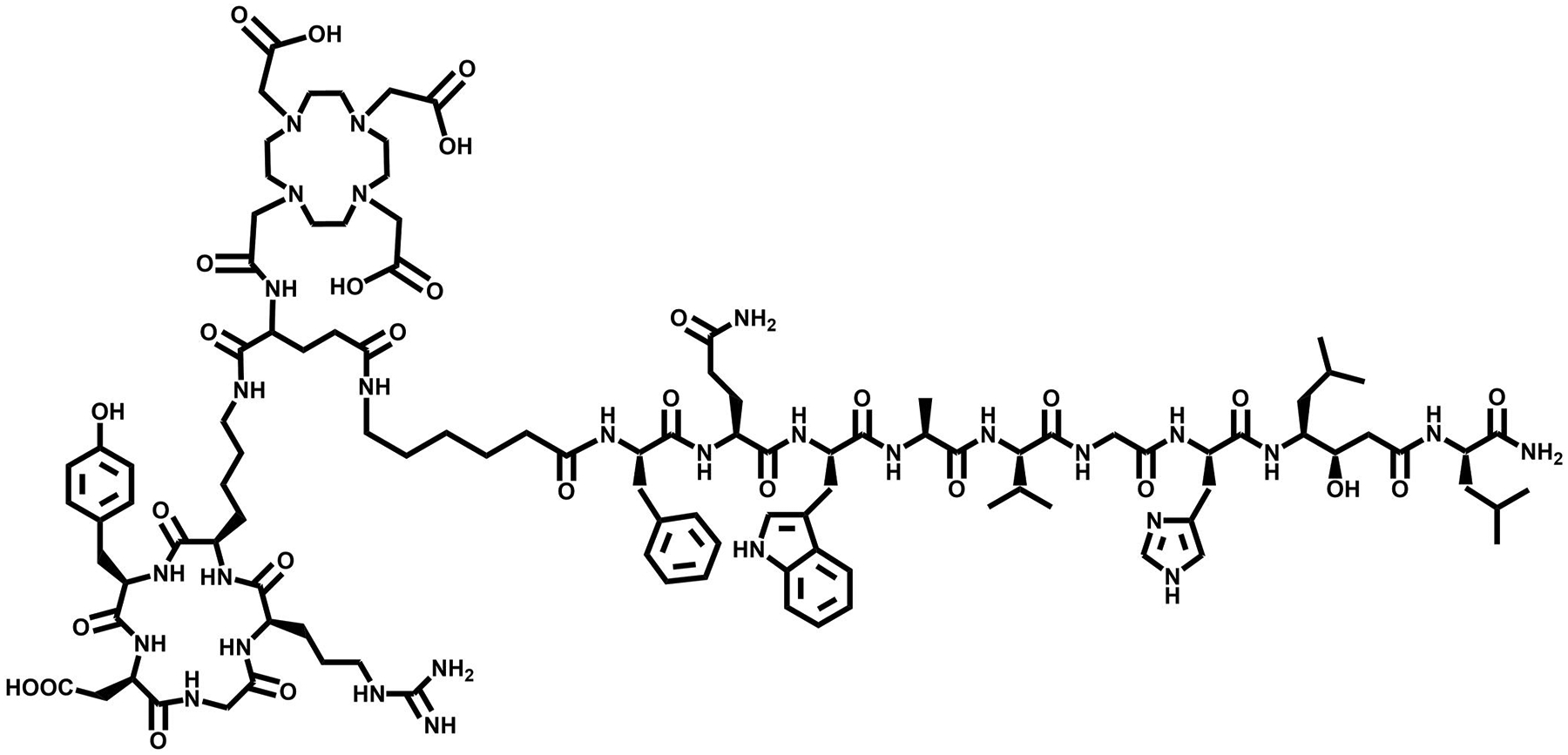
Structure of RGD-Glu-(DO3A)-6-Ahx-RM2, a heterobivalent α_v_β_3_-GRPR targeting peptide.

Stott-Reynolds et al. synthesized and evaluated heterodimeric DOTA conjugate, [RGD-Glu-(DO3A)-6-Ahx-RM2], with the aim of the study being to investigate its potential as a theranostic agent for primary and metastatic PCa (Figure 9) [96]. Metalated analogs, [RGD-Glu-[^111^In-DO3A]-6-Ahx-RM2] and [RGD-Glu-[^177^Lu-DO3A]-6-Ahx-RM2], were purified with high yields (>95%) using RP-HPLC. Studies showed high stability for both radioconjugates, showing only a minor variation from the starting material at the 24 h time point. The group believes that this is due to mild, radiolytic degradation. In vitro competitive binding assays were performed with two cell lines, PC-3, and U87-MG, using the unmetaled [RGD-Glu-(DO3A)-6-Ahx-RM2] conjugate and its metalated analogs. Both the unmetalated and conjugate metalated conjugates produced standard sigmoidal curves, depicting a dose-dependent response. The overall IC_50_ values demonstrated effective displacement of the competing radioligands indicating effective binding to both the GRPR and α_v_β_3_ targets. High affinity was shown by displacement of ^125^I-[Tyr^4^]-BBN within PC-3 Cells and moderate affinity was shown by displacement of ^125^I-Echistatin on α_v_β_3_ integrin U87-MG cells. A direct comparison between the metalated and the unmetalated compound via competitive displacement assay IC_50_ values in GRPR-expressing PC3 cells was completed. [RGD-Glu-(DO3A)-6-Ahx-RM2] (9.26 ± 0.01 nM) was compared to the metalated conjugates [RGD-Glu-(^nat^In-DO3A)-6-Ahx-RM2] (5.39 ± 1.37 nM) and [RGD-Glu-(^nat^Lu-DO3A)-6-Ahx-RM2] (5.83 ± 3.22 nM). The integrin binding affinity with the U87-MG cells demonstrated the unmetalated conjugate to have an affinity of 321 ± 82 nM in comparison to [RGD-Glu-(^nat^In-DO3A)-6-Ahx-RM2] (372 ± 22.8 nM) or [RGD-Glu-(^nat^Lu-DO3A)-6-Ahx-RM2] (346 ± 53 nM) in the same cell line [96]. Other investigator’s exploration using U87-MG cells to evaluate monovalent and bivalent conjugates have shown comparable displacement. Biodistribution investigations of [RGD-Glu-(DO3A)-6-Ahx-RM2] using normal CF-1 mice showed rapid clearance of tracer from the blood with only 0.07 ± 0.03%ID/g of the ^111^In-labeled compound and 0.06 ± 0.06%ID/g of the 177Lu-labeled compound remaining in whole blood by the 4 h time point. These results were seen to be an improvement over the [RGD-Glu-[^64^Cu-NO2A]-6-Ahx-RM2] antagonist mentioned previously. Notable pancreatic uptake in tumor bearing mice was elevated in comparison to normal mice. However, it was followed by a sharp washout by 24 h. This held a similar trend in comparison to [RGD-Glu-[^64^Cu-NO2A]-6-Ahx-RM2] [5] previously reported. Monovalent RM2 radioconjugates are known to display this phenomenon due to GRP receptors elevated expression in murine pancreas. In PC3 xenografted SCID mice, [RGD-Glu-(^111^In-DO3A)-6-Ahx-RM2] remained stable in vivo and demonstrated high retention within the tumor (1 h: 7.02 ± 0.36, 4 h: 6.67 ± 1.05, 24 h: 5.05 ± 0.10%ID/g), with minimal uptake in normal tissues. [RGD-Glu-(^111^Lu-DO3A)-6-Ahx-RM2] comparably had elevated tumor retention (1 h: 7.77 ± 0.25, 4 h: 7.05 ± 0.31, 24 h: 3.77 ± 0.28%ID/g) [96]. To ensure specificity of the tumor, mice were injected with a known blocking agent (BBN or RGD) 15 min prior to the radiotracer. In both cases, there was an approximate 80% decrease in pancreatic radio-uptake and a 36%–48% decrease in tumor uptake, demonstrating the strong affinity of these compounds for tissues expressing GRPR. The RGD blocking agent produced varying results, exhibited notable inhibitory effects on α_v_β_3_ expression in certain mice, while displaying minimal or negligible blocking impact in others. This was due, presumably, to substantially less blocking agent injected than typically used [93, 94]. Small animal SPECT/CT diagnostic images with PC-3 xenografts provided high quality images that provided excellent contrast with minimal tracer in non-tumor tissues at 20 h p.i. This indicated both radiolabeled compounds have high specificity and affinity at GRPR and α_v_β_3_ expressing sites. Future α_v_β_3_-GRPR bivalent theranostic agents have the potential to serve as promising diagnostic or therapeutic agents for primary and metastatic PCa. Research described herein warrants further investigation to explore alternative chelator/peptide combinations that may optimize the pharmacokinetic profiles of these theranostic agents to enhance clinical utility.

**FIGURE 10:**
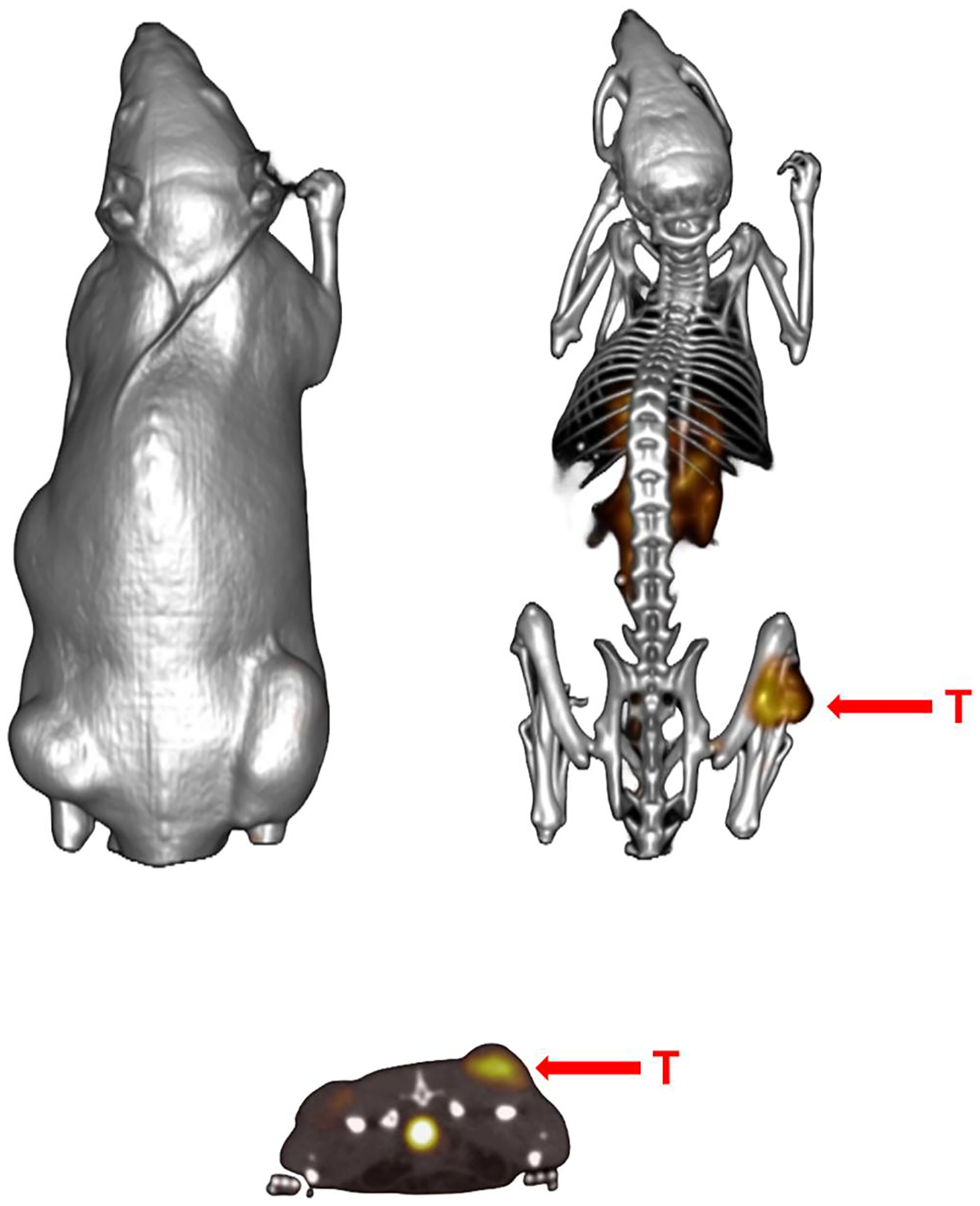
Maximum intensity small animal single-photon emission tomography (SPECT) (tumor) and CT (skeletal) fusion coronal whole-body images of PC-3 tumor bearing SCID mouse after 18 h tail vein injection of [67Ga-DO3A]-BBN ANT-RGD] heterodimer. [105] Tumor uptake at the 1 h time-point was 10.86%ID/g with retention of 4.09%ID/g at 24 h p.i. Tumors are denoted by red arrows.

The Missouri research group demonstrated the potential of α_V_β_3_-/GRPR-targeting bivalent radiotracers through their investigation of dual-targeting DOTA conjugates, namely [RGD-Glu-[^177^Lu-DO3A]-6-Ahx-RM2] and RGD-Glu-[^111^In-DO3A]-6-Ahx-RM2] [96, 105]. These conjugates exhibited exceptional success by displaying high specificity and affinity for the GRPR and α_V_β_3_ receptors (Figure 10). Notably, the study emphasized that antagonists might offer advantages over agonists for molecular imaging. However, there is a need for further research to optimize the pharmacokinetic profiles of these promising theranostic agents. Nonetheless, the successes of these radiotracers hold promise for enhancing the efficacy of current PCa treatments and serves as an exemplary model for researchers exploring diverse forms of cancer therapy.

### 3.2 | α_v_β_3_-MC1R targeting radiotracers

Malignant melanoma continues to be the most lethal form of skin cancer with an ever-increasing disease incidence rate. The cancer statistics estimate that 97,610 new cases were diagnosed, and 7990 deaths occurred in the United States in 2023 [106]. Both the melanocortin-1 receptor (MC1R) and the α_v_β_3_ integrin receptor are attractive molecular targets for melanoma due to their over-expressions on melanoma cells [78–83]. Initially, radiolabeled alpha-melanocyte-stimulating hormone (α-MSH) peptides were used to target MC1Rs for melanoma imaging [84–90], whereas radiolabeled RGD peptides have been utilized to targeting the α_v_β_3_ integrin receptors for melanoma detection [107–112], respectively. Subsequently, the over-expressions of MC1R and α_v_β_3_ integrin receptor on melanoma led to the development of radiolabeled RGD-conjugated α-MSH hybrid peptides as dual-receptor-targeting imaging probes for melanoma imaging.

**FIGURE 11:**
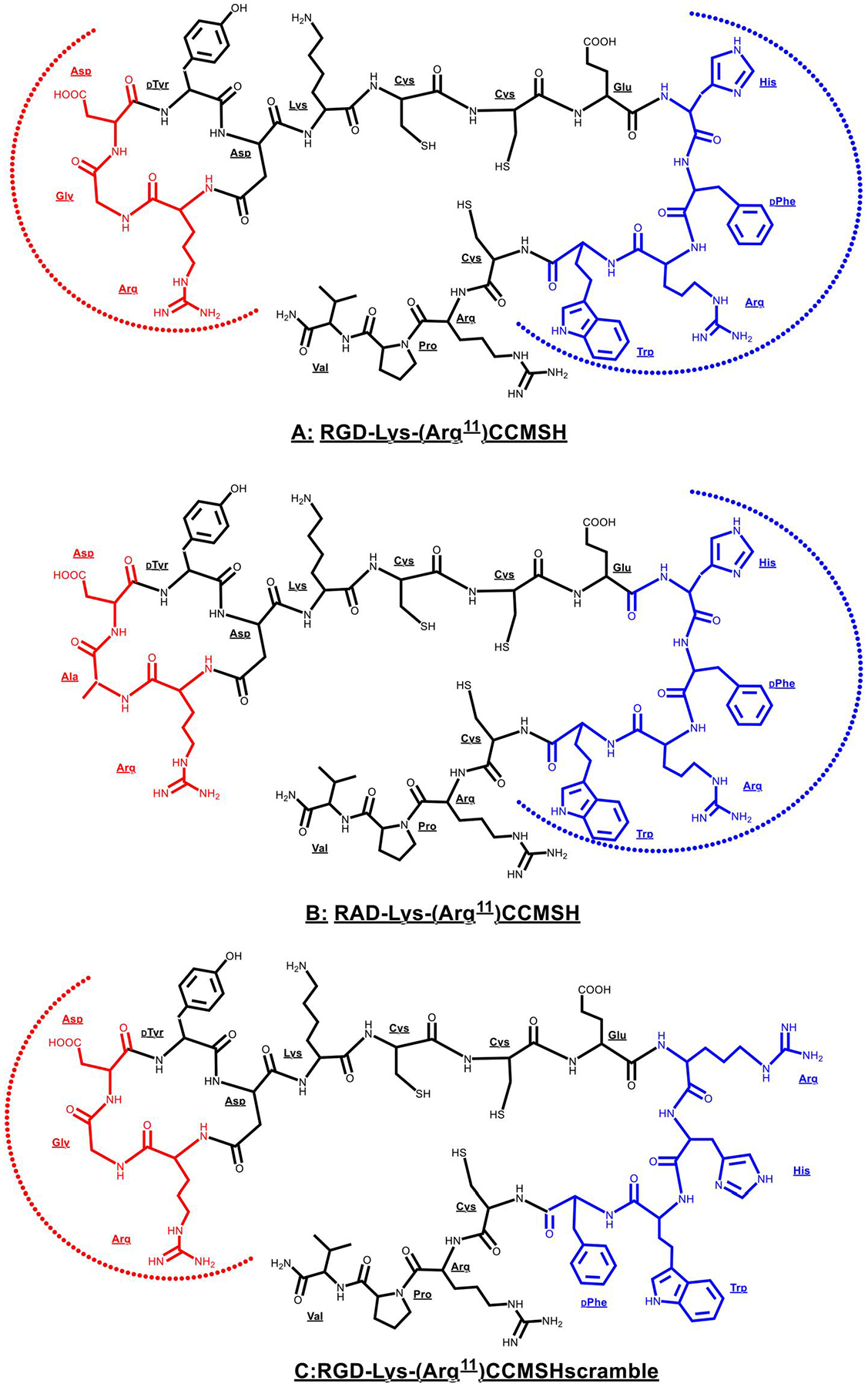
Schematic structures of RGD-Lys-(Arg^11^)CCMSH (a), RAD-Lys-(Arg^11^)CCMSH (b) and RGD-Lys-(Arg^11^)CCMSH_scramble_ (c) hybrid peptides. The receptor binding sequences were highlighted with dashed half circles. Reproduced with permission from ref. [114].

As shown in Figure 11, Yang et al. designed three hybrid peptides with similar molecular weights to demonstrate the dual-receptor-targeting property of an RGD-conjugated α-MSH hybrid peptide [113, 114]. Specifically, the cyclic RGD motif was coupled to the (Arg^11^) CCMSH through a Lys linker to generate RGD-Lys-(Arg^11^)CCMSH to target both MC1 and α_v_β_3_ integrin receptors. Meanwhile, the Gly of the cyclic RGD motif was replaced by Ala to yield RAD-Lys-(Arg^11^)CCMSH to target the MC1R only, whereas the MC1R-targeted sequence of His-DPhe-Arg-Trp of the (Arg^11^)CCMSH moiety was scrambled to the Arg-His-Trp-DPhe sequence to generate the α_v_β_3_-targeted only RGD-Lys-(Arg^11^) CCMSH_scramble_. They determined the MC1 and α_v_β_3_ integrin receptor densities on M21 human melanoma cells and revealed that 1281 MC1 receptors/cell and 96,555 α_v_β_3_ integrin receptor/cell presented on M21 human melanoma cells [114], making the M21 human melanoma xenografts a suitable animal model to examine the dual-receptor-targeting capacity of a ^99m^Tc-labeled RGD-conjugated α-MSH hybrid peptide.

The receptor binding affinities of RGD-Lys-(Arg^11^)CCMSH, RAD-Lys-(Arg^11^)CCMSH and RGD-Lys-(Arg^11^)CCMSH_scramble_ peptides clearly supported their peptide design. The dual-receptor-targeted RGD-Lys-(Arg^11^)CCMSH exhibited 2.0 and 403 nM binding affinities to the MC1 and α_v_β_3_ integrin receptors [114], respectively. Meanwhile, RAD-Lys-(Arg^11^)CCMSH maintained its 0.3 nM MC1 receptor binding affinity, but dramatically lost its α_v_β_3_ integrin receptor binding affinity by greater than 248-fold compared to RGD-Lys-(Arg^11^)CCMSH. As they anticipated, RGD-Lys-(Arg^11^)CCMSHscramble maintained its 504 nM α_v_β_3_ integrin receptor binding affinity but lost its MC1 receptor binding affinity by more than 100-fold compared to RGD-Lys-(Arg^11^)CCMSH [114]. The bio-distribution results of ^99m^Tc-labeled RGD-Lys-(Arg^11^)CCMSH, RAD-Lys-(Arg^11^)CCMSH and RGD-Lys-(Arg^11^)CCMSH_scramble_ in M21 human melanoma xenografts also strongly supported their peptide design. ^99m^Tc-RGD-Lys-(Arg^11^)CCMSH exhibited higher M21 melanoma uptake than ^99m^Tc-RAD-Lys-(Arg^11^)CCMSH or ^99m^Tc-RGD-Lys-(Arg^11^)CCMSH_scramble_. The M21 melanoma uptake value of ^99m^Tc-RGD-Lys-(Arg^11^)CCMSH was 2.5 and 2.2 times the tumor uptake value of ^99m^Tc-RAD-Lys-(Arg^11^)CCMSH and ^99m^Tc-RGD-Lys-Arg^11^)CCMSH_scramble_ [114], respectively.

Blocking studies also support the dual-receptor-targeting property of ^99m^Tc-RGD-Lys-(Arg^11^)CCMSH. Either RGD or (Arg^11^)CCMSH peptide co-injection could block 42% and 57% of the M21 melanoma uptake of ^99m^Tc-RGD-Lys-(Arg^11^)CCMSH, whereas the co-injection of RGD + (Arg^11^)CCMSH peptide mixture could block 66% of the tumor uptake of ^99m^Tc-RGD-Lys-(Arg^11^) CCMSH [114]. Moreover, the M21 melanoma uptake value of ^99m^Tc-RGD-Lys-(Arg^11^)CCMSH was higher than the sum of the melanoma uptake values of ^99m^Tc-RAD-Lys-(Arg^11^)CCMSH and ^99m^Tc-RGD-Lys-(Arg^11^)CCMSH_scramble_, indicating a synergistic (beyond additive) effect between both receptors in M21 human melanoma for ^99m^Tc-RGD-Lys-(Arg^11^)CCMSH [114]. The potential synergistic effect between the MC1 and α_v_β_3_ integrin receptors might promote the elevation of the regional peptide concentration of ^99m^Tc-RGD-Lys-(Arg^11^) CCMSH in the proximity of the MC1 and α_v_β_3_ integrin receptors. ^99m^Tc-RGD-Lys-(Arg^11^)CCMSH bound to either MC1 or α_v_β_3_ integrin receptor would be in close proximity of other available MC1 or α_v_β_3_ integrin receptors, facilitating further binding of ^99m^Tc-RGD-Lys-(Arg^11^)CCMSH molecule somewhat dissociated from the current binding receptor to other available MC1 or α_v_β_3_ integrin receptors in close proximity.

Despite the dual-receptor-targeting property of ^99m^Tc-RGD-Lys-(Arg^11^)CCMSH in M21 human melanoma-xenografted nude mice, ^99m^Tc-RGD-Lys-(Arg^11^)CCMSH exhibited remarkably high non-specific renal uptake (67.06 ± 16.53%ID/g at 2 h p.i.) [114]. Co-injection of L-Lysine reduced the renal uptake of ^99m^Tc-RGD-Lys-(Arg^11^)CCMSH by 52% at 2 h p.i. without affecting tumor uptake, suggesting that the overall positive charge of ^99m^Tc-RGD-Lys-(Arg^11^)CCMSH substantially contributed to its non-specific renal uptake. As shown in Figure 11, three Arg residues and one Lys linker represent the positive charges of ^99m^Tc-RGD-Lys-(Arg^11^)CCMSH. The Arg residues are critical to the MC1 or α_v_β_3_ integrin receptor binding. To reduce the non-specific renal uptake, Xu et al. replaced the positively charged Lys linker of ^99m^Tc-RGD-Lys-(Arg^11^)CCMSH with neutral 8-aminooctanoic acid (Aoc) or polyethylene glycol (PEG_2_) to yield ^99m^Tc-RGD-Aoc-(Arg^11^)CCMSH and ^99m^Tc-RGD-PEG_2_-(Arg^11^)CCMSH [115]. Interestingly, the bio-distribution results in M21 melanoma xenografts revealed that the substitution of the Lys linker with Aoc and PEG_2_ linker significantly reduced the renal uptake of ^99m^Tc-RGD-Aoc-(Arg^11^)CCMSH and ^99m^Tc-RGD-PEG_2_-(Arg^11^) CCMSH by 58% and 63% at 2 h p.i. The renal uptake of ^99m^Tc-RGD-Aoc-(Arg^11^)CCMSH and ^99m^Tc-RGD-PEG_2_-(Arg^11^)CCMSH was 27.93 ± 3.98 and 22.01 ± 9.89%ID/g at 2 h p.i. Meanwhile, ^99m^Tc-RGD-Aoc-(Arg^11^)CCMSH displayed similar M21 melanoma uptake as ^99m^Tc-RGD-Lys-(Arg^11^)CCMSH at 2 h p.i [115].

**FIGURE 12:**
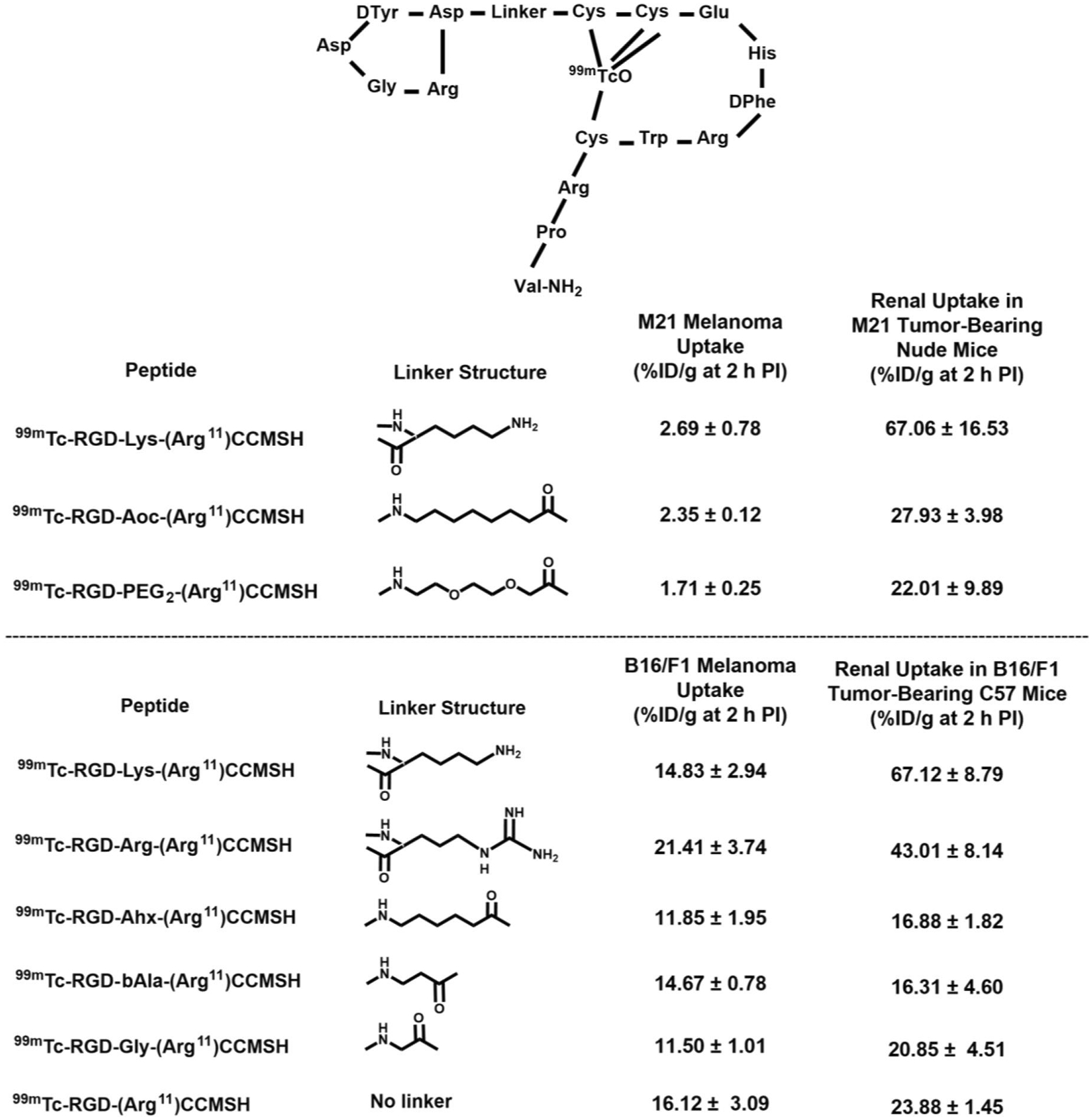
The melanoma and renal uptake of ^99m^Tc-RGD-Linker-(Arg^11^)CCMSH peptides at 2 h post-injection in M21 human melanoma-xenografted nude mice and B16/F1 murine melanoma-bearing C57 mice.

Xu et al. further examined the effect of the linker [Lys, Arg, Aminohexanoic acid (Ahx), βAla, Gly] on the bio-distribution properties of ^99m^Tc-RGD-Linker-(Arg^11^) CCMSH peptides in B16/F1 melanoma-bearing C57 mice [116–118]. Interestingly, as shown in Figure 12, the replacement of Lys with Arg dramatically reduced the renal uptake of ^99m^Tc-RGD-Arg-(Arg^11^)CCMSH by 36% at 2 h p.i. as compared to ^99m^Tc-RGD-Lys-(Arg^11^)CCMSH [116]. Moreover, the replacement of Lys with Ahx, βAla and Gly tremendously decreased the renal uptake of ^99m^Tc-RGD-Ahx-(Arg^11^)CCMSH, ^99m^Tc-RGD-βAla-(Arg^11^)CCMSH and ^99m^Tc-RGD-Gly-(Arg^11^)CCMSH by 75%, 76% and 69% at 2 h p.i. as compared to ^99m^Tc-RGD-Lys-(Arg^11^)CCMSH, respectively [114, 117]. The elimination of Lys also reduced the renal uptake of ^99m^Tc-RGD-(Arg^11^)CCMSH by 64% at 2 h p.i. as compared to ^99m^ Tc-RGD-Lys-(Arg^11^)CCMSH. The elimination or replacement of the positively charged Lys linker with neutral hydrocarbon linkers partially shielded the electrostatic interaction between the positively charged peptide molecules and negatively charged tubule cells, leading to a reduction in renal accumulation.

While the switch from RGD to RAD in RGD-Lys-(Arg^11^)CCMSH decreased the α_v_β_3_ integrin receptor binding affinity by 248-fold, surprisingly, the switch from RGD to RAD dramatically enhanced the MC1 receptor binding affinity of RAD-Lys-(Arg^11^)CCMSH as compared to RGD-Lys-(Arg^11^)CCMSH (0.3 vs. 2.0 nM) in M21 melanoma cells [114]. It is worthwhile to note that only MC1 receptors (rather than α_v_β_3_ integrin receptors) are overexpressed on B16/F1 cells [114]. Therefore, the selection of the B16/F1 melanoma model could minimize the contribution of α_v_β_3_ integrin receptors to the melanoma uptake of dual receptor-targeting ^99m^Tc-RGD-Lys-(Arg^11^)CCMSH. Interestingly, such improvement in the MC1 receptor binding affinity eventually increased the B16/F1 melanoma uptake of ^99m^Tc-RAD-Lys-(Arg^11^) CCMSH as compared to ^99m^Tc-RGD-Lys-(Arg^11^)CCMSH. The B16/F1 tumor uptake of ^99m^Tc-RAD-Lys-(Arg^11^) CCMSH was 1.5, 1.3 and 1.4 times the tumor uptake of ^99m^Tc-RGD-Lys-(Arg^11^)CCMSH at 0.5, 2 and 4 h p.i., respectively [119].

The structural difference between ^99m^Tc-RAD-Lys-(Arg^11^)CCMSH and ^99m^Tc-RGD-Lys-(Arg^11^)CCMSH is Ala and Gly. Specifically, Ala has one more methyl group than Gly. Such minor structural differences from Gly to Ala resulted in stronger MC1R binding affinity and higher B16/F1 melanoma uptake for ^99m^Tc-RAD-Lys-(Arg^11^) CCMSH [119]. It was desirable to understand how the replacement of Gly with other amino acids could affect the melanoma targeting and pharmacokinetic properties of ^99m^Tc-RXD-Lys-(Arg^11^)CCMSH peptides. Therefore, the Gly was replaced by a variety of amino acids including Thr, Val, Ser, Nle, Phe and DPhe to yield a class of RXD-Lys-(Arg^11^)CCMSH peptides [120, 121]. The introduction of Thr, Val, Ser, Nle, Phe and DPhe generated different impact on the MC1 receptor binding affinities of the peptides on B16/F1 melanoma cells. The MC1R binding affinities of the peptides ranged from 0.7 to 3 nM. Among these six peptides, RTD-Lys-(Arg^11^)CCMSH displayed the strongest MC1R binding affinity (0.7 nM), whereas RNleD-Lys-(Arg^11^)CCMSH exhibited the weakest MC1R binding affinity (3 nM). Although the -His-DPhe-Arg-Trp-motif is the binding moiety to MC1 receptor, the difference in receptor binding affinity suggested that the amino acid at this position subtly interacted with the receptor binding moiety. Such subtle interactions were likely related to the flexibility of lactam bonds among amino acid residues in the peptides.

^99m^Tc-RTD-Lys-(Arg^11^)CCMSH, ^99m^Tc-RVD-Lys-(Arg^11^)CCMSH and ^99m^Tc-RSD-Lys-(Arg^11^)CCMSH exhibited comparably high receptor-mediated B16/F1 melanoma uptake to ^99m^Tc-RAD-Lys-(Arg^11^)CCMSH. However, ^99m^Tc-RVD-Lys-(Arg^11^)CCMSH and ^99m^Tc-RSD-Lys-(Arg^11^)CCMSH displayed similar lower renal uptake than ^99m^Tc-RTD-Lys-(Arg^11^)CCMSH by approximately 30% at 0.5, 2, and 4 h p.i [120, 121]. Not surprisingly, ^99m^Tc-RFD-Lys-(Arg^11^)CCMSH and ^99m^Tc-RfD-Lys-(Arg^11^)CCMSH showed much higher liver and renal up-take than ^99m^Tc-RSD-Lys-(Arg^11^)CCMSH. Despite the high B16/F1 melanoma uptake associated with some ^99m^Tc-RXD-Lys-(Arg^11^)CCMSH peptides, it was desirable to address the issue of high renal uptake of ^99m^Tc-RXD-Lys-(Arg^11^)CCMSH peptides (67–135%ID/g at 2 h p.i.). Co-injection of L-lysine dramatically decreased the renal up-take of ^99m^Tc-RTD-Lys-(Arg^11^)CCMSH, ^99m^Tc-RVD-Lys-(Arg^11^)CCMSH and ^99m^Tc-RSD-Lys-(Arg^11^)CCMSH by 40%–50%, suggesting that the overall positive charges of the ^99m^Tc-RXD-Lys-(Arg^11^)CCMSH peptides played key roles in their non-specific renal uptake.

The substitution of the positively charged Lys linker with a neutral βAla yielded three ^99m^Tc-RXD-βAla-(Arg^11^)CCMSH peptides, whereas the further replacement of positively charged Arg in RAD moiety generated three ^99m^Tc-XAD-βAla-(Arg^11^)CCMSH peptides [122]. The substitution of the substitution of the positively charged Lys linker with a neutral βAla reduced the overall positive changes of ^99m^Tc-RXD-βAla-(Arg^11^) CCMSH peptides. Meanwhile, the modification of positively charged Arg in the RAD moiety further decreased the overall positive charge of ^99m^Tc-XAD-βAla-(Arg^11^) CCMSH peptides. The substitution of the Lys linker with a βAla linker resulted in the reduction in tumor uptake for ^99m^Tc-RXD-βAla-(Arg^11^)CCMSH peptides by 21%–45% in B16/F1 melanoma-bearing C57 mice at 2 h p.i. as compared to ^99m^Tc-RAD-Lys-(Arg^11^)CCMSH [121, 122]. Specifically, the tumor uptake of ^99m^Tc-RAD-βAla-(Arg^11^)CCMSH (15.66 ± 6.19%ID/g) was 79% of the tumor uptake of ^99m^Tc-RAD-Lys-(Arg^11^)CCMSH at 2 h p.i. (11, 12). The substitution of the Lys linker with a β-Ala linker dramatically decreased the renal uptake of ^99m^Tc-RXD-β-Ala-(Arg^11^)CCMSH peptides by 64%–79% in B16/F1 melanoma-bearing C57 mice at 2 h p.i. as compared to ^99m^Tc-RAD-Lys-(Arg^11^)CCMSH. The renal uptake of ^99m^Tc-RAD-βAla-(Arg^11^)CCMSH (20.18 ± 3.86%ID/g) was 22% of the tumor uptake of ^99m^Tc-RAD-Lys-(Arg^11^) CCMSH at 2 h p.i. Co-injection of 15 mg of L-lysine further reduced the renal uptake of ^99m^Tc-RAD-βAla-(Arg^11^)CCMSH from 20.18 ± 3.86%ID/g to 13.06 ± 3.62% ID/g at 2 h p.i. without significantly affecting the tumor uptake. However, the replacement of Arg in ^99m^Tc-RAD-βAla-(Arg^11^)CCMSH with Nle and Glu did not further decrease the renal uptake [122].

**FIGURE 13:**
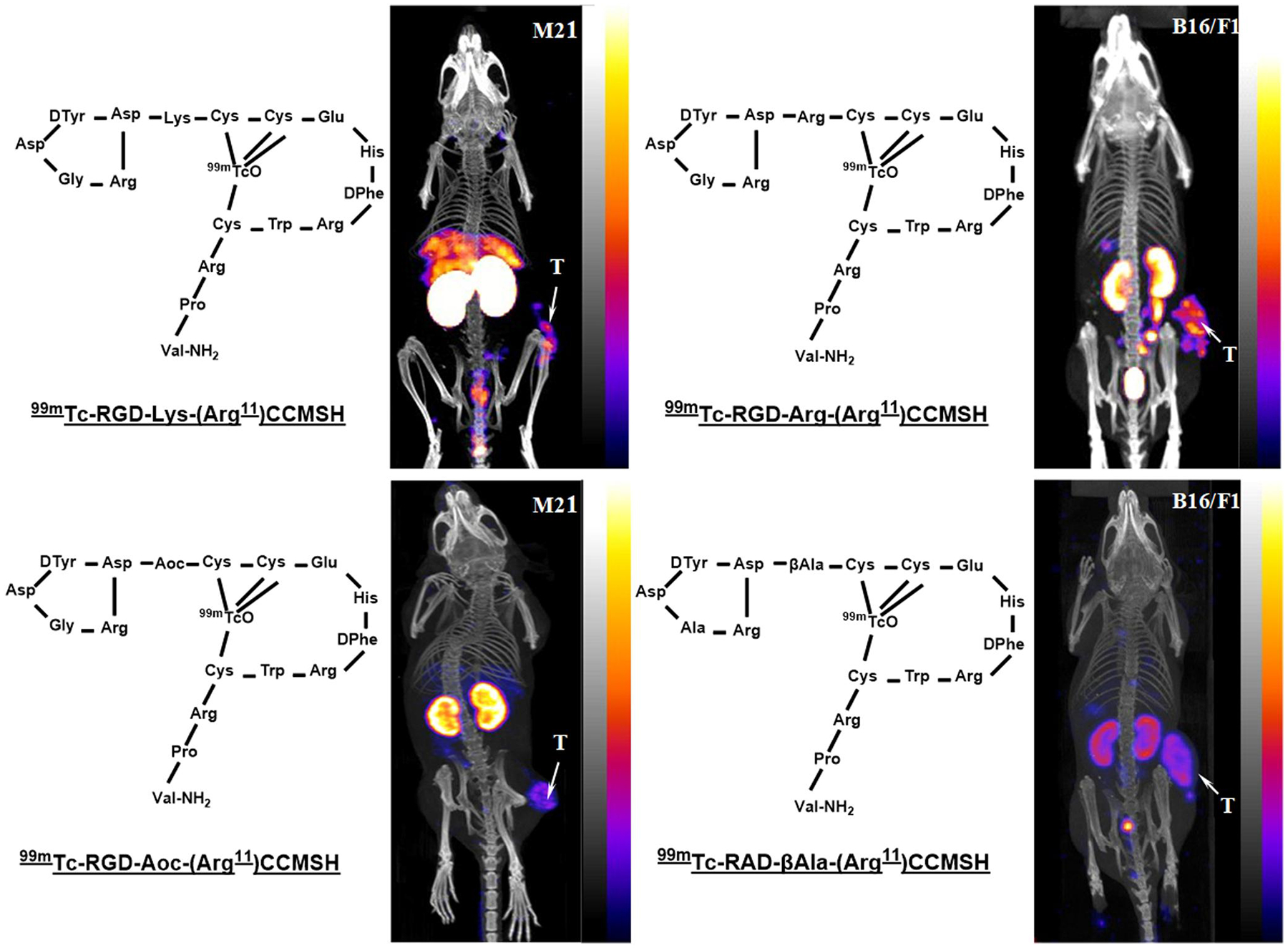
Maximum intensity projection single-photon emission tomography (SPECT)/CT images of ^99m^Tc-RGD-Lys-(Arg^11^) CCMSH and ^99m^Tc-RGD-Aoc-(Arg^11^)CCMSH on M21 human melanoma-xenografted nude mice (left), ^99m^Tc-RGD-Arg-(Arg^11^)CCMSH and ^99m^Tc-RAD-βAla-(Arg^11^)CCMSH on B16/F1 melanoma-bearing C57 mice (right) at 2 h post-injection, respectively. Reproduced with permission from ref. [114, 115, 116, 122].

The representative SPECT/CT images of ^99m^Tc-RGD-Lys-(Arg^11^)CCMSH and ^99m^Tc-RGD-Aoc-(Arg^11^)CCMSH on M21 human melanoma-xenografted nude mice, ^99m^Tc-RGD-Arg-(Arg^11^)CCMSH and ^99m^Tc-RAD-βAla-(Arg^11^)CCMSH on B16/F1 melanoma-bearing C57 mice are summarized in Figure 13. Both M21 and B16/F1 melanomas could be clearly visualized and the effect of linker on the renal uptake were demonstrated in small animal SPECT images (Figure 13).

Both receptor density and receptor binding affinity are crucial factors to consider in the development of an imaging probe targeting two receptors (MC1 and α_v_β_3_ integrin receptors) overexpressed on M21 human melanoma cells. Potential synergistic effect between two receptors and the delicate balance in dynamic ligand-receptor binding play roles in the success of a dual receptor-targeting peptide as well. Despite the relatively low MC1 receptor density (1281 receptors/cell) and high nanomolar α_v_β_3_ integrin receptor binding affinity (403 nM), high α_v_β_3_ integrin receptor density (96,555 receptor/cell) and low nanomolar MC1 receptor binding affinity (2.0 nM) might substantially contribute to the successful visualization of M21 human melanoma using ^99m^Tc-RGD-Lys-(Arg^11^)CCMSH as an imaging probe. Presumably, higher MC1R density and stronger α_v_β_3_ integrin receptor binding affinity would further enhance the success of dual-receptor-targeted radiolabeled peptides for melanoma detection (Table 3).

### 3.3 | α_v_β_3_-SST2 cell-targeting radiotracers

A critical component of tumoral growth and development involves angiogenesis. Researchers have grown interested in the possibility of regulating angiogenesis in hopes of developing an improvement to modern-day cancer therapy. Most of the current research pertains to targeting cancer cells through monovalent conjugates. The De Jong group is one of the very few groups striving to enhance the pharmacokinetic profiles of potential drugs against cancer through bivalent methodologies [123–125]. Using a combination of two distinct sequences of α_v_β_3_ targeting RGD and SST2 targeting analogs, the group believes a dual targeting heterodimer could have therapeutic potential. SST2 receptors, significantly upregulated on various cancer cell surfaces, notably those of NE origin, remain unresponsive to conventional therapeutic regimens including chemotherapy and external beam radiation. α_v_β_3_ receptors exhibit a similar upregulation. Combining an agent to target both receptors simultaneously and leveraging the synergistic effects of an apoptosis-inducing factor, such as the α_v_β_3_-targeting RGD motif, may amplify radiotherapeutic efficacy across a spectrum of cancers.

**FIGURE 14:**
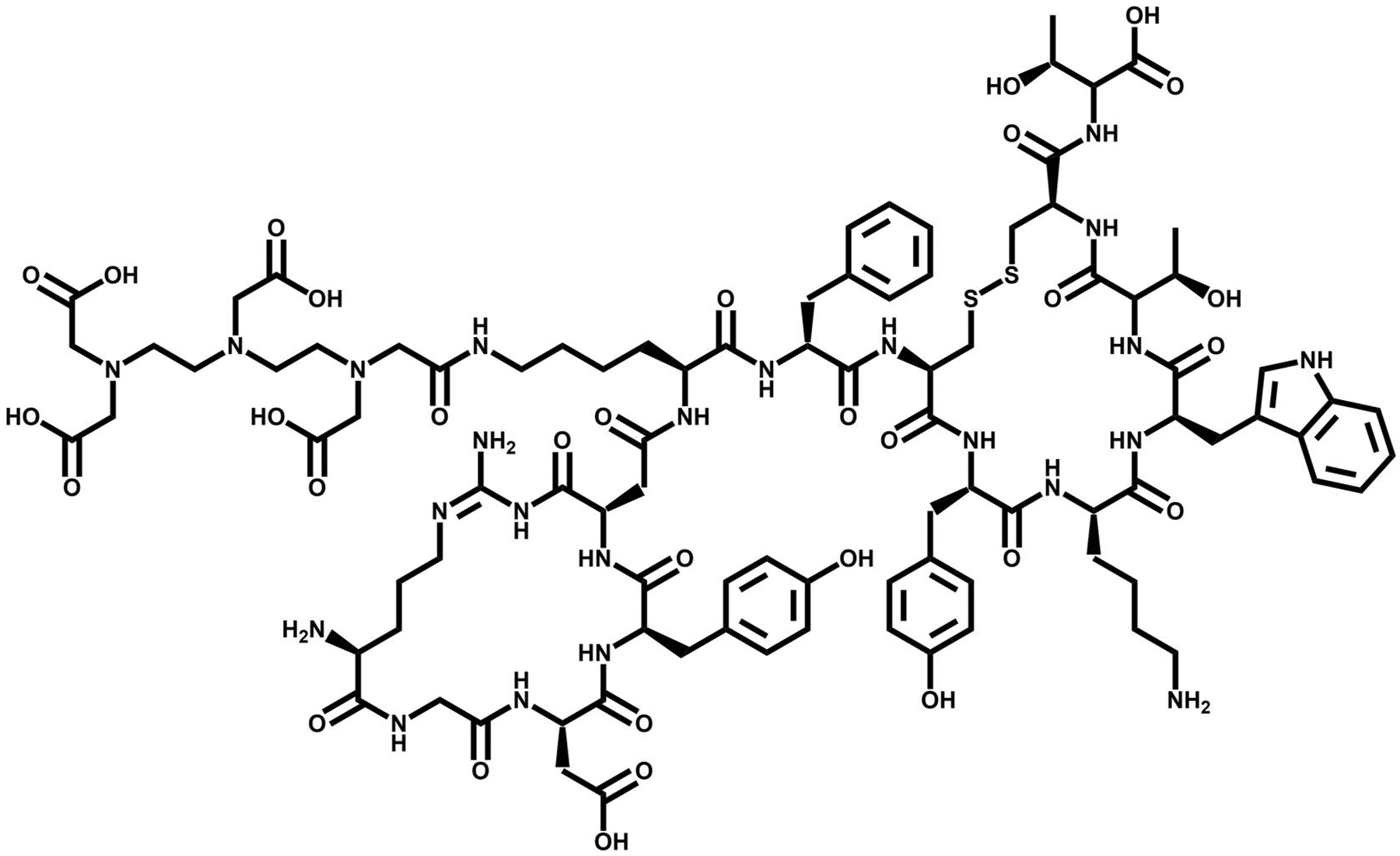
Structure of RGD-DTPA-Tyr3-octreotate, a heterobivalent αvβ3-SST2 targeting vector.

One of the initial reports of a SST-α_v_β_3_ bivalency approach was by Bernard et al. [123], featuring the bivalent peptide, RGD-DTPA-Tyr^3^-octreotate (Figure 14). This conjugate comprises three main components: RGD for inducing apoptosis potential, SST targeting agent Tyr^3^-octreotate for enhanced internalization, and the bifunctional chelator DTPA for radiolabeling. RGD-DTPA-Tyr^3^-octreotate was radiolabeled with 111In and evaluated for specificity in vivo and in vitro. The in vitro internalization in SST2 positive CA20948 tumor cells of the radiolabeled hybrid analogue appeared to be a rapid process, blockable by excess unlabeled octreotide, indicating an SST2-specific mechanism. Biodistribution results of RGD-^111^In-DTPA-Tyr^3^-octreotate in rats xenografted with CA20948 or α_v_β_3_-positive AR42J tumors agreed with the uptake and internalization data. The bivalent radiotracer demonstrated a tumor uptake like that of monovalent radiolabeled octreotate in vivo but demonstrated high renal uptake as well, only partially blockable after injecting octreotide. Pharmacokinetic properties of RGD-^111^In-DTPA-Tyr^3^-octreotate were directly compared to RGD-^111^In-DOTA-Tyr^3^-octreotate, revealing higher renal radiometal accumulation in the kidney for the DTPA conjugate in comparison to the DOTA conjugate [123]. While the radiolabeled bivalent peptide showed high tumor uptake and retention, the substantial renal accumulation makes this drug impractical for future theranostic application. To mitigate this, the effect of injecting D-lysine along with the radiotracers to reduce kidney uptake was evaluated. Analyses included the co-injection of 400 mg/kg of D-lysine to determine the possibility of decreasing renal uptake. Results show a reduction of RGD-^111^In-DTPA-Tyr^3^-octreotate by 40% within the renal system [123].

Capello et. al. [124] further investigated this bivalent analogue through in vitro assays to analyze cell death activity. Colony forming assays employing various cell lines were used to evaluate tumoricidal effects based on apoptosis, linked with caspase-3 activity. The bivalent RGD-^111^In-DTPA-Tyr^3^-octreotate conjugate exhibited significantly increased apoptosis compared to either ^111^In-RGD or ^111^In-DTPA-Tyr^3^, both of which had minimal effect on cell survival [124]. Due to high renal uptake, the bivalent analogue is deemed inappropriate for therapeutic use at this stage of development (Table 4). However, unlabeled RGD-DTPA-octreotate continues to induce apoptosis for tumor-induced angiogenesis which indicates therapeutic potential for this compound [125].

### 3.4 | α_v_β_3_-PSMA cell-targeting radiotracers

Prostate Specific Membrane Antigen and integrin α_v_β_3_, are biomarkers that are overexpressed on the cell surface of certain tumor epithelium as well as most solid tumor neovasculature and are critical targets for cancer therapy. Their overexpression in a variety of malignancies makes them attractive targets for simultaneous targeting, enhancing affinity toward numerous cancer types across different patient cohorts. The Pomper lab has developed a heterobivalent agent, EUKL-cRGDfK-NH_2_ functionalized with either a near-infrared dye (IRDye800CW) or bifunctional chelator DOTA for theranostic applications [126]. These agents demonstrate multitargeting capabilities by incorporating a low molecular weight PSMA-targeting urea-based PSMA inhibitor and a cyclic RGD (cRGDfK) α_v_β_3_-targeting moiety with a β-glutamic acid linker into a single construct. In vitro assays utilizing near-infrared cell-based binding assays involving PSMA-positive PC3-PIP and α_v_β_3_-expressing U87-MG cells were conducted to assess the binding affinities of the conjugated heterobivalent probe. The results showed comparable affinities to their respective monovalent compounds, validating the efficacy of the construct. Subsequent in situ ligand energy minimization algorithm experiments were completed to predict the binding mode of the DOTA-conjugated heterobivalent agent. These computational analyses supported a model describing the conformations adopted by the agent, enabling its binding to both protein targets. Subsequently, in vivo optical assays were conducted using EUKL-cRGDfK-NH2-IRDye800 on PC3-PIP, U87-MG, and PC3 (negative control) xenografted mice. These studies demonstrated high uptake in both PSMA and α_v_β_3_ malignancies, and the uptake was effectively blockable using a respective cell line-specific blocking agent, affirming the targeting specificity of the heterobivalent agent. These findings collectively underline the promising potential of EUKL-cRGDfK-NH_2_ for targeted imaging and therapy in diverse cancer contexts, highlighting its potential clinical translational value.

### 3.5 | Prostate specific membrane antigen-gastrin-releasing peptide receptors cell-targeting radiotracers

Since 2014, overall incidence rates of PCa, the second most leading cancer in men, have been increasing by roughly 3% annually [127]. For advanced-stage disease, a 5% increase has been observed each year with only a 32% 5 year survival for patients with metastatic PCa. In 2023, it is estimated that there will be 34,700 male fatalities attributed to metastatic disease, with a mortality rate of 18.8% among affected patients [106]. GRPR and PSMA are promising molecular targets for PCa radiopharmaceuticals due to their heightened expression on PCa cells. Monovalent species of PSMA-targeting radiotracers are successful and available for clinical use, while RM2 GRPR-targeting tracers are awaiting FDA approval. Despite their success, the inherent heterogeneity of tumors presents a challenge for increasing the specificity of monovalent targeted radiotracers. Gastrin Releasing Peptide Receptor expression is elevated in earlier stages of PCa, while PSMA is upregulated in locoregional or late-stage disease [128]. An investigation directly comparing the performance of ^68^Ga-RM2, a GRPR-targeted radiotracer, and two different drugs, a PSMA-targeted agent, revealed that 43 lesions in only 18 of the 50 patients could be detected by one of the radiotracers. This culminated in the development of a single heterobivalent conjugated radiotracer targeting both GRPR and PSMA to effectively target multiple bio-markers, thereby enhancing its applicability in the detection, staging, and treatment of both primary and metastatic PCa.

**FIGURE 15:**
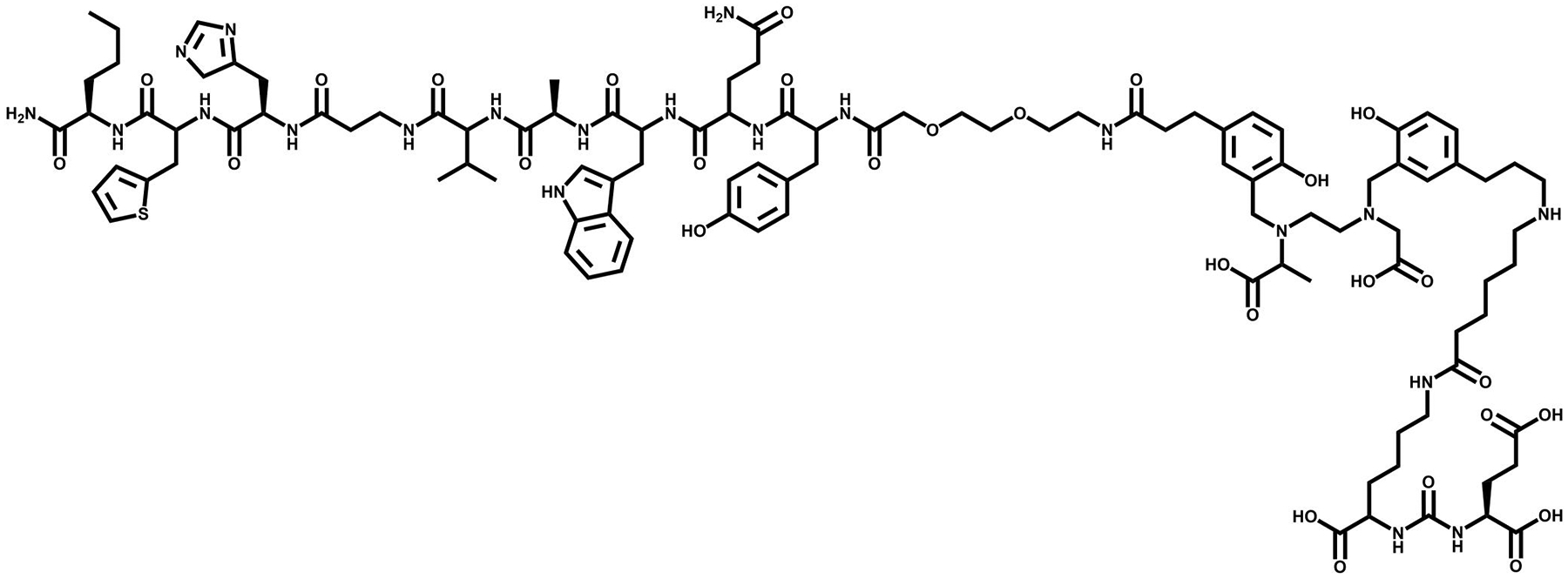
Structure of Glu-urea-Lys(Ahx)-HBED-CC-BZH3, a heterobivalent PSMA-GRPR targeting vector.

Studies investigating PSMA-BBN-based heterodimers have been documented and have produced diverse outcomes (Table 5). Eder et al. [129] have prepared, via fmoc-solid phase peptide synthesis, a urea-based PSMA inhibitor with the nonapeptide BZH3 for GRPR targeting to yield the compound Glu-urea-Lys(Ahx)-HBED-CC-BZH3 (Figure 15). This bispecific molecule was radiolabeled with ^68^Ga and showed strong binding affinity to both PSMA and GRPR in competitive displacement as-says (25.0 ± 5.4 nM and 9.0 ± 1.8 nM, respectively) as well as pharmacokinetic uptake and excretion closely resembling their respective monomeric building blocks [129]. In biodistribution investigations, GRPR+ AR42J xenografted mice showed uptake of 5.4 %ID/g and PSMA+ LNCaP bearing mice showed 3.3 %ID/g accumulation in tumors with high PSMA-mediated uptake values in the kidneys and spleen. Subsequently, pharmacokinetic properties of this molecule were optimized by incorporating hydrophilic linkers between the HBED chelator and the BXH3 GRPR-targeting moiety [134]. The three amino acid linkers tested, (HE)_n_ (*n* = 0–3) were made up of positively charged His and negatively charged Glu to decrease the relatively high radiotracer uptake in the kidneys and spleen. The results of this modified low-molecular weight heterodimer HE_1–3_ showed decreased liver and spleen accumulation and also a reduction in kidney uptake (>50%) in comparison to the first-generation radiotracer [129, 134].

A multipurpose, bivalent [DUPA-6-Ahx-(NODAGA)-5-Ava-BBN(7–14)NH_2_] radioconjugate labeled with ^64^Cu for PCa imaging was developed by the Smith research lab in 2014 [4]. Specifically, urea-based PSMA targeting DUPA ((2-[3-(1,3-dicarboxypropyl)-ureido]pentanedioic acid))and GRPR targeting BBN(7–14)NH_2_ were synthesized via solid-phase peptide synthesis, followed by the manual conjugation of a NODAGA chelator to the ε amine of lysine. Cyclotron produced ^64^Cu is an attractive radiometal for PET molecular imaging because of its ideal nuclear properties, making it useful in vivo. In vitro assays including fluorescence microscopy and competitive binding were performed to ensure specifi-city and selectivity. Fluorescence microscopy of a Rhodamine-B derivative conducted at 590 nm demonstrated the successful targeting and biomarker specificity for both GRPR and PSMA expressing cells using a single radioligand. Small animal PET molecular images acquired using [DUPA-6-Ahx-(^64^Cu-NODAGA)-5-Ava-BBN(7–14)NH2] illustrated the efficacy of the radiotracer in detecting PCa. Binding affinity for LNCaP homogenized cell membranes (PSMA positive) were investigated using a N-acetylated-α-linked acidic dipeptidase (NAALDase) assay and revealed high receptor binding affinity for the [DUPA-6-Ahx-(^nat^Cu-NODAGA)-5-Ava-BBN(7–14)NH2] conjugate (IC_50_ = 1.16 ±1.35 nM) [4].GRPR binding in PC3 cells was evaluated via competitive displacement binding assays and showed an IC_50_ of 3.09 ± 0.34 nM. Subsequently, biodistribution analyses in PC-3 xenografted SCID mice were conducted demonstrating predominate renal clearance, and notable retention of the radioligand in the liver, spleen, small intestine, and PSMA-positive kidney. Larger molecular size and hydrophobic properties may have contributed to this uptake and subsequent retention of tracer. The substantial accumulation and retention of the tracer in the gut limits the ability of [DUPA-6-Ahx-(^64^Cu-NODAGA)-5-Ava-BBN(7–14)NH_2_] to target primary and metastatic lower abdominal disease. The PET images acquired for [DUPA-6-Ahx-(^64^Cu-NODAGA)-5-Ava-BBN (7–14)NH2] in PC-3 (GRPR-positive) and LNCaP (PSMA-positive) PCa cells did not exhibit greater performance when compared to analogous monovalent counterparts [4].

**FIGURE 16:**
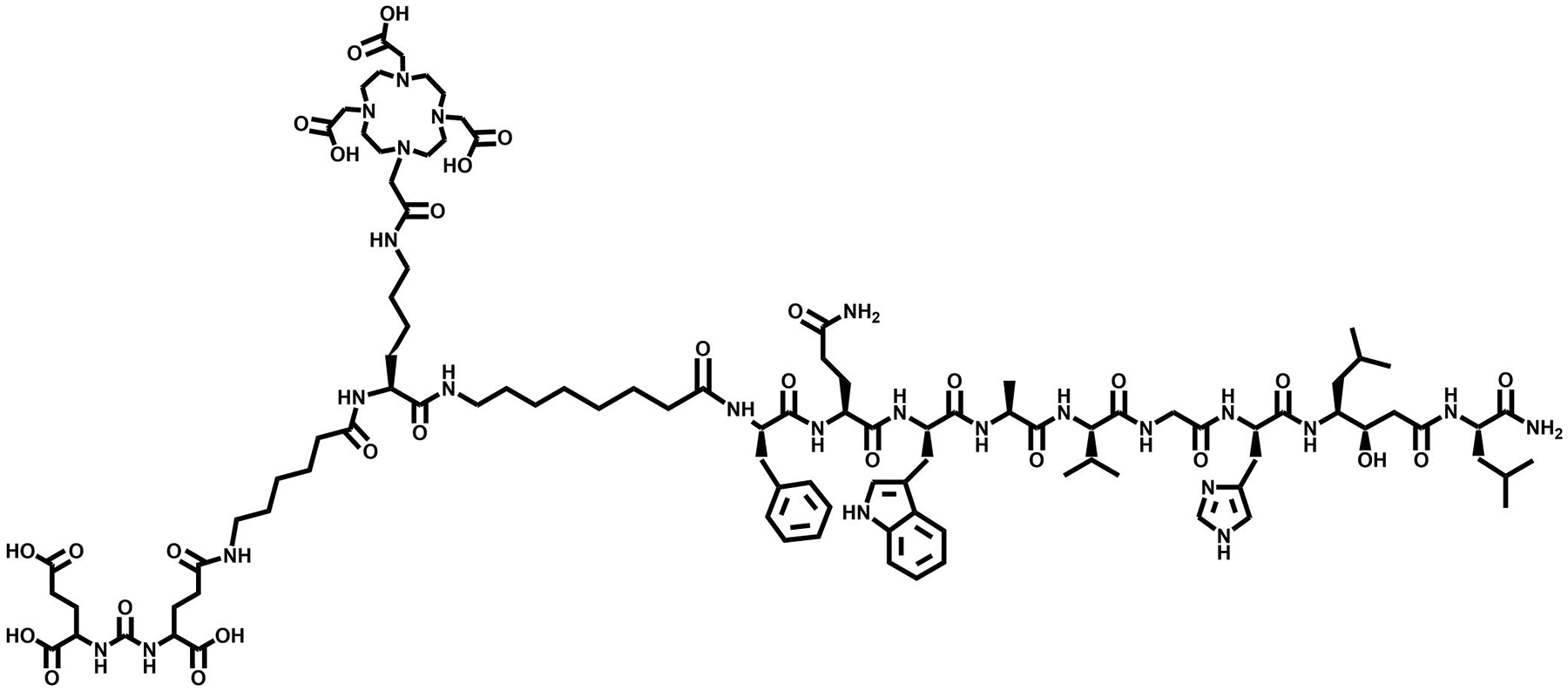
Structure of [DUPA-6-Ahx-Lys(DOTA)-6-Ahx-RM2], a heterobivalent PSMA-GRPR targeting vector.

**FIGURE 17:**
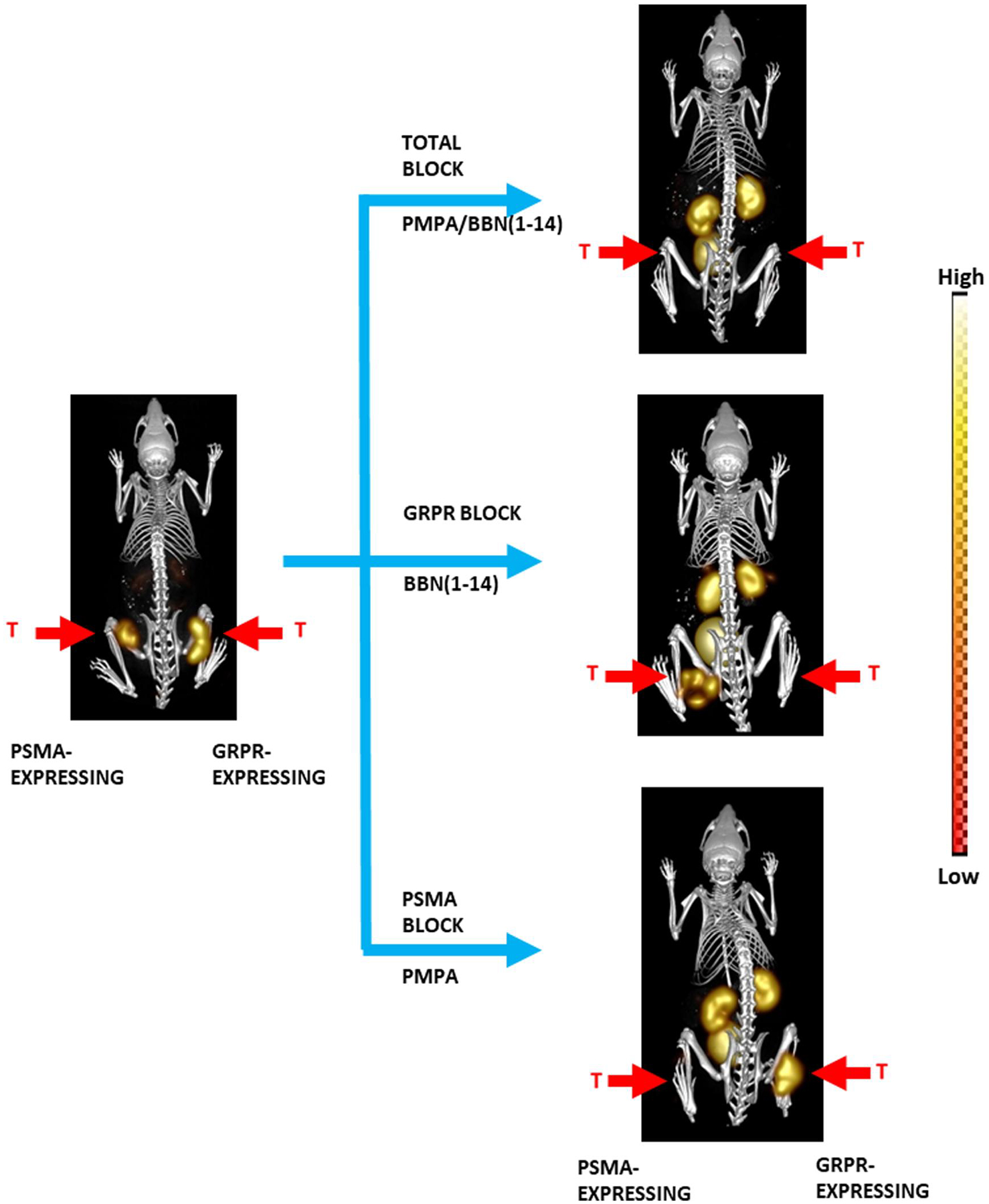
[DUPA-6-Ahx-(DOTA)-8-Aoc-BBN ANT] radiolabeled with ^111^In in PC-3 and PC-PIP tumor-bearing SCID mice at 4 h post-tail vein injection of bilateral xenografted tumors. Whole-body images are maximum-intensity microSPECT registered to microCT. The ^111^In labeled conjugate had uptake values of 4.74 ± 0.90%ID/g in PC3 tumors and 5.38 ± 1.07%ID/g in PC3-PIP tumors. Tumors are denoted by red arrows.

As a result of these studies, this same research team moved forward to create an alternative PSMA-BBN agent to decrease background activity in collateral organs by changing from agonist BBN(7–14)NH_2_ to an antagonist GRPR-targeting moiety. Work by Bandari et al. Progressed further in the enhancement of a superior dual-biomarker targeting ligand, [DUPA-6-Ahx-Lys(DOTA)-6-Ahx-RM2], as part of their ongoing development efforts (Figure 16). This targeting agent maintained the urea based PSMA targeting motif and modified the GRPR motif to the antagonist RM2. Following synthesis and characterization of [DUPA-6-Ahx-Lys(DOTA)-6-Ahx-RM2], the bivalent agent was metalated with ^nat/67^GaCl_3_, ^nat/111^InCl_3_, and ^nat/177^LuCl_3_ with high radiochemical purity and yield (≥95%). Competitive displacement assays with LNCaP and PC-3 human cancer cell lines demonstrated high binding affinities for PSMA and GRPR of 9.30 ± 2.32 nM and 3.99 ± 1.8 nM, respectively [135]. The 6-aminohexanoic acid pharmacokinetic modifier was substituted with 8-aminooctanoic acid to form the novel [DUPA-6-Ahx-Lys (DOTA)-8-Aoc-RM2]. Thereafter, the bivalent species was metalated in high radiochemical yield (≥98%) with ^nat/111^InCl_3_ for SPECT imaging and ^nat/177^LuCl_3_ for possible TRT [3]. This compound showed improved pharmacokinetic and biodistribution results in comparison to the first-generation radiotracer. At 1 h p.i., accumulation of [DUPA-6-Ahx-Lys(^177^Lu-DOTA)-8-Aoc-RM2] in PC3 and PC3-PIP xenografted mice were 7.51 ± 2.61 and 7.37 ± 2.89%ID/g, respectively. The ^111^In labeled conjugate had uptake values of 4.74 ± 0.90%ID/g in PC3 tumors and 5.38 ± 1.07%ID/g in PC3-PIP tumors. Single-photon emission tomography/CT investigations at 4 h p.i. showed no uptake in collateral tissues other than kidney, with high uptake and retention in tumors [3]. A blocking investigation was completed to validate the multivalent nature of the tracers. Mice bearing bilateral PSMA- and GRPR-expressing PC3-PIP and GRPR-expressing PC3 tumors were administered a blocking agent of either 2-PMPA or BBN(1–14) peptide as well as a total block administering both agents 15 min prior to the injection of the radiotracer (Figure 17).

The research team at the National Autonomous University of Mexico have evaluated their own BBN/PSMA-targeting bivalent radiotracer with the BBN agonist Lys^3^-BN in efforts to decrease radiotracer uptake in benign tissues as demonstrated previously [131]. The ^68^Ga radiolabeled bivalent, Glu-CO-Lys[2Nal-Cys [Lys^3^(GMBS)-BBN-NH_2_]-DOTA] (denoted iPSMA-BN), demonstrated high stability in human serum up to 3 h and greater cell uptake and internalization in both PC3 and LNCaP cells in comparison to ^68^Ga-iPSMA or ^68^Ga-BN. Pharmacokinetic properties showed PSMA mediated high spleen and renal uptake with rapid excretion via the renal-urinary system. Preclinical imaging done with PC3 and GRPR expressing tumors in the lungs of nude mice demonstrated favorable uptake in PCa lesions [131]. This agent was subsequently administered intravenously to four healthy volunteers (2 male, 2 female) to determine the biokinetics and dosimetry in human patients [132]. The blood clearance was rapid at T_1/2_ = 2.64 min in comparison to the monomer ^68^Ga-iPSMA (T_1/2_ = 6.5 min in the blood). The internal iliac nodes were clearly visualized (SUV_max_ = 4.7) due to targeted radiotracer accumulation. However, the monovalent species ^68^Ga-iPSMA showed a higher SUV_max_ than the^68^Ga-iPSMA-BN heterodimer in the same tissue [132].

The PSMA/GRPR specific heterodimer, NOTA-DUPA-RM26, prepared by Mitran et al. [133] has been synthesized and radiolabeled using both ^111^In and ^68^Ga.This bivalent radioligand displayed high stability and favorable specific binding affinity to PSMA and GRPR in vitro. This was demonstrated through experiments using PSMA-expressing LNCaP cells, GRPR-expressing PC3 cells, and dual expressing PC3-PIP cells. Furthermore, the in vivo imaging outcomes revealed only partial blockage of the radiotracer at 1 h p.i., using PSMA-617 or NOTA-PEG6-RM26 as the blocking agents in PC3-PIP xeno-grafted tumor models [133].

Bailly et al. [136] have investigated a new heterodimer specific for PSMA and GRPR in the context of creating heterobivalent ligands through a simplified “one-pot” synthetic approach. This strategy seeks to mitigate the challenges associated with the arduous production of intricate dual-targeting molecules. The modular synthesis process can be accomplished within less than 24 h, utilizing a molecular scaffold with reactive 3,6-dichloro-1,2,4,5-tetrazine as the initial material and suitable partners for sequential nucleophilic aromatic substitution and inverse electron-demand Diels-Alder reactions. This reaction effectively increases the distance between the two targeting moieties from the dichlorotetrazine platform, a crucial aspect for optimizing the advantages of ligand heterobivalency. The two targeting moieties in this synthesis were the low molecular weight PSMA-targeting urea-based inhibitor KuE (lysine-urea-glutamate) and the GRPR-targeting antagonist JMV594. Evaluation of the pharmacologic properties of the 68Ga labeled radio-conjugate through in vivo and in vitro studies demonstrated that the platform-based assembly method does not hinder the interaction of the ligands with their receptors [136].

## 4 | CONCLUSION

The field of personalized nuclear medicine is currently undergoing a transformative shift due to the introduction of multimeric peptide-based radiopharmaceuticals. Although considerable progress has been achieved in recent years by conventional techniques involving monovalent bioactive peptides, nanoparticles, or antibodies, researchers are increasingly embracing a heterobivalent strategy to enhance the radiotracer’s pharmacological properties and theranostic potential. As the scientific community shifts toward heterobivalent approaches, greater leaps will be made in new drug delivery systems in the fight against cancer. Advances in radiochemistry and chelation chemistry have further allowed precise tuning of these peptides for radiolabeling with various radionuclides, catering to a wide array of medical applications within nuclear oncology. As research continues to evolve, these insights pave the way for the continued development and implementation of innovative radiopharmaceuticals into the clinical arena.

While traditional monovalent approaches have continued commendable achievements, they lack the ability to face limitations with the intricate, heterogeneous nature of tumors. Not all cancers are the same and can vary in type and amount depending on the individual patient or the stage of cancer. The bivalent radiotracers described herein provide substantial evidence in displaying the advantages of amalgamating the dual receptor-targeting actions of emerging theranostic radiopharmaceuticals. While the synthesis of heterobivalent ligands is acknowledged for its complexity and time-consuming nature, streamlined methods of synthesis are ongoing expedite the difficult process. It is believed the potential benefits of these radiopharmaceuticals outweigh the inherent challenges associated with their synthesis. By utilizing a single agent to target multiple known biomarkers, the potential impact encompasses broader patient coverage, precise staging, customized treatment design, and the potential for improved patient outcomes and quality of life.

In conclusion, employing bivalency to engage multiple targets simultaneously holds the potential to surpass the capabilities of monovalent peptide-based radiopharmaceuticals. As the scientific community gradually embraces the usage for heterobivalent approaches, successful strategies to effectively combat cancer across diverse patient profiles and varying cancer stages will be developed. Harnessing the combined potential between multiple targeting strategies, these approaches could unlock new dimensions of accuracy, sensitivity, and specificity of disease.

## Figures and Tables

**TABLE 1:** Common metal-based radionuclides for either molecular imaging (**γ** or **β**^+^) or targeted radiotherapy (TRT) (**β**^−^ or **α**).

Radionuclide	Production	Decay	Half life (T_1/2_)	E^+/−^ (MeV)	Application
^64^Cu	^64^Ni(p,n)^64^Cu	EC, β^−^, β^+^	12.7 h	**γ**: 1.3458 **β**^−^: 0.578 **β**^+^: 0.651	Diagnostic
^67^Cu	^68^Zn(γ,p)^67^Cu	β^−^, γ	61.8 h		Therapeutic
^68^Ga	^68^Ge/^68^Ga Generator	EC, β^+^	67.7 min	**γ**: 1.0773 **β**^+^: 1.899	Diagnostic
^86^Y	^86^Sr(p,n)^86^Y	EC, β^+^	14.74 h	**γ**: 1.0767, 0.6278, 1.153.1 **β**^+^: 1.248	Diagnostic
^89^Zr	^89^Y(p,n)^89^Zr	EC, β^+^, γ	78.4 h	**γ**: 0.909, 1.713, 1.657 **β**^+^: 0.897	Diagnostic
^90^Y	^90^Sr/^90^Y Generator	β^−^	64.2 h	**β**^−^: 2.27	Therapeutic
^99m^Tc	^99^Mo/^99m^Tc Generator	IT	6.1 h	**γ**: 0.1427	Diagnostic
^111^In	^112^Cd(p,2n)^111^In	EC	67.2 h	**γ**: 0.1713, 0.2454	Diagnostic
^153^Sm	^152^Sm(n,γ)^153^Sm	β^−^, γ	46.3 h	**β**^−^: 0.69, 0.64 **γ**: 0.1032, 0.0697	Therapeutic
^166^Ho	^165^Ho(n,γ)^166^Ho	β^−^, γ	26.8 h	**β**^−^: 1.855, 1.773 **γ**: 0.0806, 1.3794	Therapeutic
^177^Lu	^176^Lu(n,γ)^177^Lu	β^−^, γ	6.65 d	**β**^−^: 0.497 **γ**: 0.2084, 0.1129	Therapeutic, diagnostic
^186^Re	^185^Re(n,γ)^186^Re	β^−^, γ	3.8 d	**β**^−^: 1.071, 0.933 **γ**: 0.1372	Therapeutic
^211^At	^209^Bi(α,2n)^211^At	EC, α, γ	7.21	**γ**: 0.1372 **α**: 5.868	Therapeutic
^203^Pb	^203^Tl(p,n)^203^Pb	Γ	51.9 h	**γ**: 279.2	Diagnostic
^212^Pb	^224^Ra/^212^Pb Generator	β^−^, γ	10.6 h	**β**^−^: 0.569, 0.335 **γ**: 0.3000, 0.2386	Therapeutic

**TABLE 2:** A brief outline of α_v_β_3_-GRPR receptor-targeting radiotracers.

Compound name	Isotope	Reference number
RGD-BBN	^18^F	[93]
NOTA-RGD-BBN	^68^Ga, ^64^Cu	[97, 98]
FB-PEG_3_-Glu-RGD-BBN	^18^F	[98]
DO3A-RGD-BBN	^177^Lu	[94]
NO2A-RGD-Glu-6-Ahx-BBN (7–14)NH2	^64^Cu	[95]
RGD-Glu-[NO2A]-6-Ahx-RM2	^64^Cu	[5]
RGD-Glu-[DO3A]-6-Ahx-RM2	^111^In, ^177^Lu	[96]

**TABLE 3:** A brief outline of α_v_β_3_-MC1R receptor-targeting radiotracers.

Compound name	Isotope	Reference number
RGD-X-(Arg^11^)CCMSH	^99m^Tc	[114–118]
X = Lys, PEG_2_, Arg, Ahx, βAla, Gly		
RXD-Lys-(Arg^11^)CCMSH	^99m^Tc	[119, 121]
X = Thr, Val, Ser, Nle, Phe, DPhe		

**TABLE 4:** A brief outline of α_v_β_3_-SST2 receptor-targeting radiotracers.

Compound name	Isotope	Reference number
RGD-DTPA-Tyr^3^-octreotate	^111^In	[123]
RGD-DOTA-Tyr^3^-octreotide	^111^In	[123]

**TABLE 5:** A brief outline of PSMA-GRPR receptor-targeting radiotracers.

Compound name	Isotope	Reference number
Glu-urea-Lys(Ahx)-HBED-CC-BZH3	^68^Ga	[129]
[DUPA-6-Ahx-(NODAGA)-5-Ava-BBN (7–14)NH_2_	^64^Cu	[4]
[DUPA-6-Ahx-Lys(DOTA)-6-Ahx-RM2]	^67^Ga, ^111^In, ^177^Lu	[130]
[DUPA-6-Ahx-Lys(DOTA)-8-Aoc-RM2]	^111^In, ^177^Lu	[3]
Glu-CO-Lys[2Nal-Cys[Lys^3^(GMBS)-BBN-NH_2_]-DOTA] (iPSMA-BN)	^68^Ga	[131, 132]
NOTA-DUPA-RM26	^111^In, ^68^Ga	[133]

## Data Availability

There is no data for this review.

## Abbreviations:

α: alpha
α-MSH: α-Melanocyte-Stimulating Hormone
β^−^: beta
β^+^: positron
γ: gamma
BBN: bombesin peptide
BFCA: bifunctional chelating agent
GRPR: gastrin-releasing peptide receptors
MC1R: melanocortin 1 receptor
NE: neuroendocrine
NEC: neuroendocrine carcinoma
NET: neuroendocrine tumors
p.i.: post-intravenous injection
PCa: prostate cancer
PEG: polyethylene glycol
PET: positron emission tomography
PRRT: peptide receptor radionuclide therapy
PSMA: prostate specific membrane antigen
RAFT: regioselective addressable functionalized template
SPECT: single photon emission computed tomography
SST: somatostatin
TRT: targeted radionuclide therapy
